# Physical stimuli-responsive CRISPR-Cas9 systems for spatiotemporally precise control of genome engineering

**DOI:** 10.7150/thno.122238

**Published:** 2026-01-01

**Authors:** Jinbin Pan, Bingjie Li, Yaqiong Wang, Yating Han, Guijun Liu, Shao-Kai Sun

**Affiliations:** 1 Department of Radiology, Tianjin Key Laboratory of Functional Imaging, Tianjin Medical University General Hospital, Tianjin 300052, China.; 2 Athinoula A. Martinos Center for Biomedical Imaging, Institute for Innovation in Imaging (i 3 ), Department of Radiology, Massachusetts General Hospital and Harvard Medical School, Boston, MA 02129, USA.; 3 Department of Radiology, Zhongshan Hospital, Fudan University, Shanghai 200032, China.; 4 Department of Cell Biology, School of Basic Medical Sciences, Tianjin Medical University, Tianjin 300070, China.; 5 School of Medical Imaging, Division of Medical Technology, Tianjin Key Laboratory of Functional Imaging, Tianjin Medical University, Tianjin 300203, China.

**Keywords:** CRISPR/Cas9, genome engineering, spatiotemporal control, stimuli-responsive, delivery systems

## Abstract

The Clustered Regularly Interspaced Short Palindromic Repeats-associated protein 9 (CRISPR-Cas9) endonuclease system has revolutionized biology research by enabling precise, efficient, and versatile genome editing. However, achieving spatiotemporally controlled gene editing within specific organs, tissues, or cells remains a major challenge, as unregulated CRISPR-Cas9 activity can lead to severe off-target effects, hindering its clinical translation. To enhance the on-target precision and reduce the unwanted consequences of aberrant or premature CRISPR-Cas9 activation, various strategies have been developed to regulate its function at translational or post-translational stages using diverse external physicochemical stimuli. While chemical molecule-inducible CRISPR-Cas9 systems have demonstrated significant progress, most of them still suffer from inherent deficiencies, such as unsatisfactory spatiotemporal precision, irreversibility, pharmacokinetic dependence, internal disturbance, and safety concerns related to chemical inducers. By contrast, externally applied physical stimuli provide distinct advantages for triggering CRISPR-Cas9 activity, offering superior spatiotemporal precision, reversibility, and biocompatibility. These features significantly enhance the controllability, target specificity, and practical applicability of CRISPR-Cas9 systems across diverse biological settings. This review systematically explores recent advances in physical stimuli-responsive CRISPR-Cas9 platforms, detailing their design strategies, activation mechanisms, and proof-of-concept applications. Furthermore, we provide a comparative analysis of different stimulation strategies, highlighting their respective characteristics, current limitations, and future prospects. A discussion on the persistent bench-to-bedside gap is also included, aiming to guide future development toward clinically viable solutions.

## 1. Introduction

Clustered Regularly Interspaced Short Palindromic Repeats (CRISPR)-associated protein 9 (Cas9) system, as a transformative gene-editing platform, has revolutionized gene regulation and therapeutic strategies by enabling precise, efficient, and convenient modification of target genes. The CRISPR-Cas system was originally discovered in bacteria and archaea as a form of adaptive immunity providing protection against phage attacks 1-3. To date, CRISPR-Cas systems have been classified into 2 classes, 6 types, and nearly 40 subtypes 4-6, with the type II CRISPR-Cas9 system from *Streptococcus pyogenes* being the most extensively studied and widely utilized for genome editing 7.

**Figure 1:**
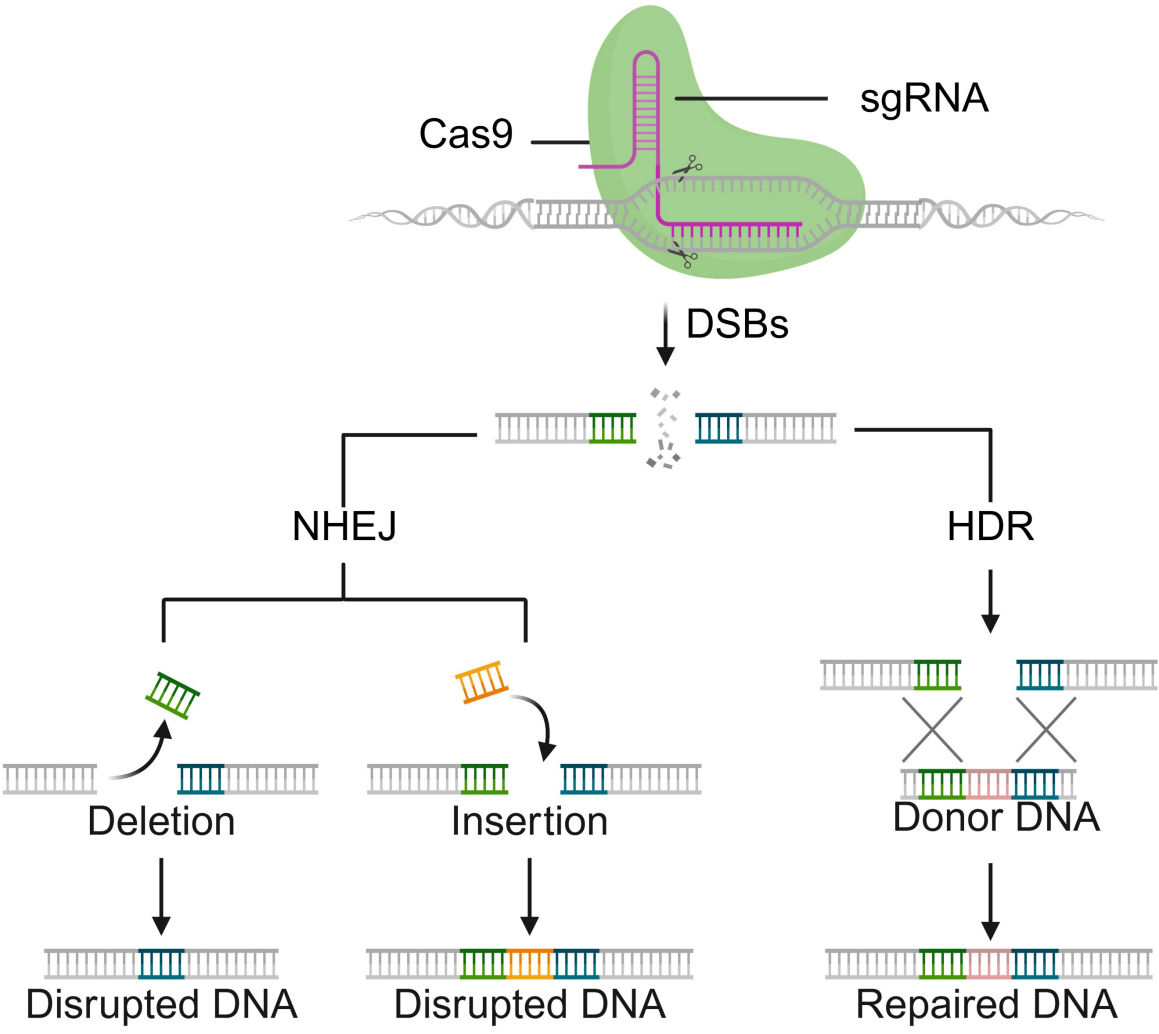
Schematic illustration of sgRNA-guided Cas9 nuclease for DSBs, followed by endogenous repairing pathways: NHEJ or HDR pathways.

Naturally occurring Cas9 proteins (orthologs) contain two nuclease domains, namely the RuvC- and HNH-like domains, which can cleave exogenous DNA to form a double-strand break (DSB) after recognizing a distinct protospacer adjacent motif (PAM) (**Figure 1**) 8. Cleavage is directed by two RNAs: the CRISPR RNA (crRNA), which confers target specificity, and the trans-activating crRNA (tracrRNA), which enables crRNA maturation; these can be fused into a single-guide RNA (sgRNA) to streamline genome editing. Following DNA cleavage, the DSB of DNA is repaired via either the nonhomologous end-joining (NHEJ) pathway, leading to insertion/deletion (indel) mutations, or the homology-directed repair (HDR) pathway, which facilitates accurate sequence insertion when a donor DNA template is available 9-11. Notably, mutating either the HNH or RuvC-like domain yields a nickase (nCas9), whereas dual mutations (D10A and H840A in SpCas9) generate a catalytically inactive, RNA-guided DNA-binding protein (dCas9) that retains targeting without cleavage 7. Unlike other site-specific nuclease platforms such as zinc-finger nucleases (ZFNs) 12, transcription activator-like effector nucleases (TALENs) 13, and meganucleases 14, the CRISPR-Cas9 system uses the same protein scaffold for different targets, with specificity dictated by a short, easily retargeted sgRNA, avoiding complex protein engineering. Given the remarkable merits of versatility, flexibility, cost-effectiveness, and easy engineering, the CRISPR-Cas9 system is becoming a highly adaptable tool not only for genome-editing studies but also for broader applications in genome and chromatin manipulation 15, 16. For example, catalytically impaired dCas9 supports targeted transcriptional and epigenetic modulation as well as chromatin visualization/topology studies, whereas nCas9-based fusions underlie high-efficiency base editing and prime editing without introducing DSB 17-19. Collectively, the advent and widespread adoption of the CRISPR-Cas9 system with unprecedented simplicity and flexibility have ushered in a new era of genome engineering 20.

Although the CRISPR-Cas9 system can achieve high editing efficiencies, reaching up to ~90% indels in optimized cellular contexts, it has also been criticized for off-target activity 21. Off-target effects arise when the Cas9-sgRNA complex binds and cleaves unintended genomic loci; in some settings, off-target sites can tolerate multiple mismatches relative to the intended protospacer and may even involve bulges 22, 23. From an evolutionary standpoint, a slightly relaxed specificity can be advantageous for bacterial immunity against rapidly mutating phages. Clinically, however, even low-frequency off-target mutations can be consequential, risking chromosomal rearrangements, genomic instability, and loss-of-function in essential genes 24. Beyond intrinsic sequence-driven off-targeting observed in basic research, growing attention in *ex vivo*/*in vivo* applications have focused on efficacy, safety, and cell/tissue/organ specificity 25. Especially, the treatments of numerous genetic diseases, such as Duchenne muscular dystrophy 26, Leber congenital amaurosis type 10 27, hereditary tyrosinemia 28, alpha-1 antitrypsin deficiency 29, and solid tumors 30, ultimately require the *in vivo* modification of pathogenic genes within relevant tissues. Achieving this demands efficient and selective delivery of genome-editing cargos (e.g., Cas9 ribonucleoproteins or mRNA, sgRNA, and, when applicable, donor templates/base or prime editor constructs), which remains challenging 31. Two exposure-driven risks further compound the problem: spatial off-targeting (on-target editing occurring in the wrong cells/tissues) and temporal overexposure (undesired persistent Cas9 expression after the desired edit) 32, 33. Minimizing both the intrinsic sequence-level off-target effects and these exposure-related risks is therefore central to clinical translation. Consequently, precise spatial and temporal regulation of CRISPR-Cas9 activity is essential for effective and safe *in vivo* genome editing.

To address this challenge, diverse promising approaches have been explored to mitigate the off-target effect of CRISPR-Cas9 in both basic research and *in vivo* applications, such as engineering high fidelity Cas9 variants, optimizing sgRNA design, excavating transcriptome analysis, delivering CRISPR-Cas9 machinery via functional materials, decreasing the duration of the nuclease expression, as well as exploiting physicochemical-inducible systems 4, 41. Among these strategies, chemical and physical stimuli-inducible systems can enable precise spatiotemporal regulation of Cas9 activity, effectively reducing off-target genome modifications by restricting CRISPR-Cas9 expression to target cells/tissues/organs, and its duration post-editing. Chemical-inducible CRISPR-Cas9 systems have been widely applied across various research fields, enabling precise and controllable genome editing, and their significance has been extensively summarized in several reviews 5, 42, 43, highlighting their versatility and potential for targeted therapeutic applications. Nevertheless, they still face limitations such as barely satisfactory spatiotemporal precision, irreversibility, pharmacokinetic dependence, internal disturbance, and safety concerns related to chemical inducers 5, 34, 35. These systems often rely on transcriptional regulation (e.g., Tet-On/Off or inducible Cre systems) 44, 45, which suffers from delayed response times and lacks the ability to tightly regulate Cas9 activity within a defined temporal window.

In contrast to chemical methods, external noninvasive physical stimuli (e.g., light, temperature, magnetic fields, ultrasound) have garnered increasing attention due to their high spatiotemporal precision and reversibility, making them a promising tool for minimizing off-target effects in CRISPR-Cas9 genome editing 46-48. These systems allow for the precise regulation of Cas9 activity, with much less risk of undesirable effects in non-target cells, tissues, or organs. While light-controlled CRISPR-Cas9 systems have been widely explored 42, 49-51, non-optical physical triggers such as magnetic and ultrasound have also emerged as exciting alternatives 52, offering distinct advantages for tissue-specific and deep-tissue editing. Particularly, magnetogenetic strategies enable modulation with minimal pharmacokinetic dependence 53, whereas sonogenetic approaches use focused ultrasound to confine editing to small, predefined volumes with rapid on/off control 54. **Table 1** outlines the advantages and disadvantages of chemical and physical inducible systems, highlighting the distinctive benefits of physical stimuli for gene editing. Given their emerging potential, a systematic and in-depth review specifically focusing on various physical stimuli-induced CRISPR-Cas9 systems is crucial and urgently needed for the understanding and promotion of their development.

**Figure 2:**
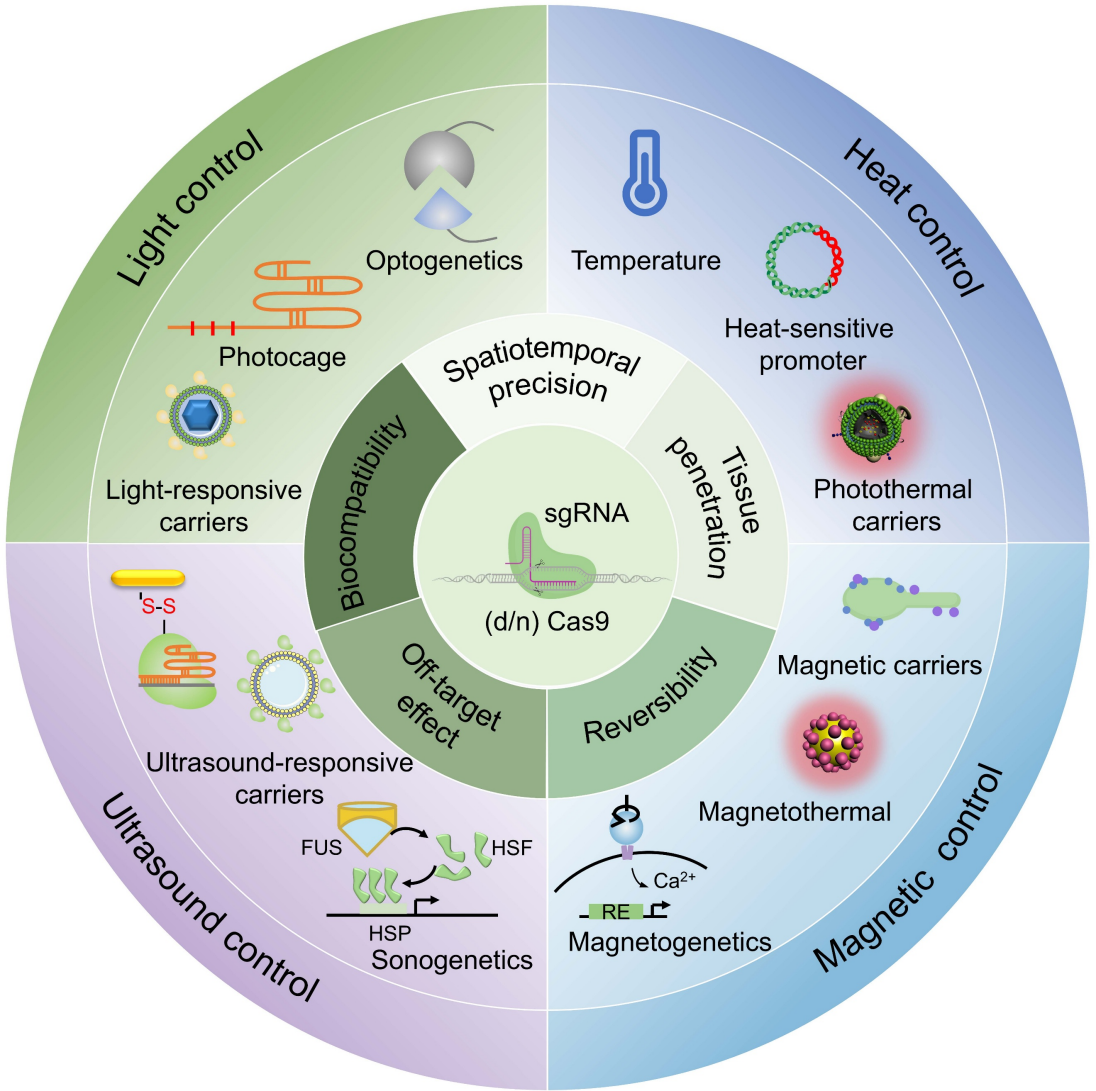
Illumination of physical stimuli-responsive CRISPR-Cas9 systems towards spatiotemporally precise control of genome engineering.

This review summarizes recent advances in physically responsive CRISPR-Cas9 systems for precision genome engineering (**Figure 2**). We systematically examine key design strategies, activation mechanisms under diverse physical stimuli, and representative proof-of-concept applications. A comparative analysis is provided to evaluate the advantages, limitations, and translational challenges across different triggering modalities. In contrast to previous reviews, which often prioritize optical control, this work offers a balanced treatment of thermal, magnetic, and ultrasonic approaches alongside optical strategies, establishing a coherent narrative from molecular mechanism to biological application. Adopting a translation-focused perspective, we assess each physical trigger using practical metrics, such as spatiotemporal precision, tissue penetration, reversibility, biocompatibility, and off-target effects, and introduce an exposure-driven risk framework to illustrate how physical actuation mitigates key biosafety concerns. Importantly, we highlight emerging non-optical strategies such as magnetogenetics and sonogenetics, which have received limited coverage in earlier reviews despite their promise for deep-tissue targeting, device compatibility, and rapid reversibility. It is our aim that this perspective will not only facilitate the understanding of design principles underlying physically controlled genome editors but also stimulate further innovation toward safe and effective *in vivo* therapeutic applications.

## 2. Physical stimuli-responsive CRISPR-Cas9 systems with spatiotemporal precision

### 2.1 Optical control

Light serves as an ideal external physical trigger for activation and deactivation of biomolecules, offering non-invasive control with high spatial and temporal resolution. Harnessing light has afforded remarkable advances in biological research and holds great promise for clinical applications. Consequently, light-mediated regulation of the CRISPR-Cas9 system, including Cas9 and its variants (dCas9 and nCas9), for spatiotemporal control of genome engineering has become highly attractive. Here, the optical CRISPR-Cas9 systems were categorized into three types based on the core components affected by light: (d/n)Cas9, sgRNA, and non-viral delivery systems.

#### 2.1.1 Optical control of (d/n)Cas9

Light affords precise spatial and temporal control of Cas9. At the protein level, several mechanistic logics predominate and can be applied to wild-type Cas9. These are irreversible photocaging, reversible protein switching by reconstitution, and indirect optical gating through inhibitors or expression. Together, they define a coherent design space for optical regulation from cells to tissues.

**Figure 3:**
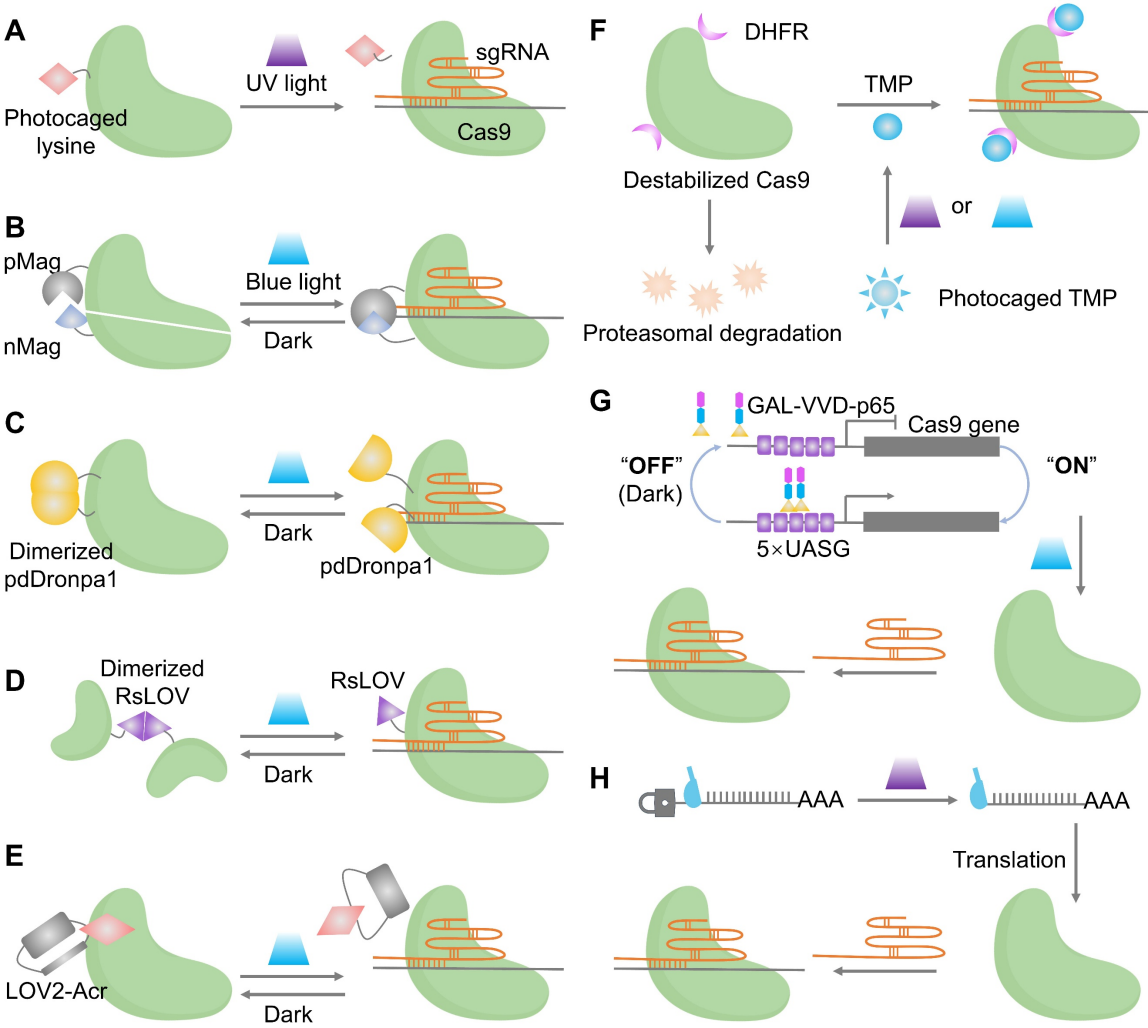
Optical control of Cas9 for genome editing. (A) The caged Cas9 protein with a photocaged lysine. (B) Photo-activable Cas9 system consisting of two split Cas9 fragments linked to light-inducible dimerization domains, pMag and nMag. (C) Cas9 system fused with light-inducible depolymerization domains of pdDronpa1. (D) Photo-switchable Cas9 system fused with light-inducible dimerization domains of RsLOV. (E) Optogenetic control of the CRISPR-Cas9 system via the light-inducible LOV2-Acr hybrids. (F) A light-inducible gene expression system utilizing CRISPR-Cas9 and the optimized, light-switchable tool GAVPO. (G) Precise control over Cas9 activity by photocaged TMP combined with DHFR domains. (H) Cas9 mRNA with a photocleavable group on the 5′ cap.

Photocaged activation establishes the temporal control by blocking an essential residue with a photolabile group that is removed by ultraviolet (UV) exposure to restore wild-type activity (**Figure 3A**). This approach is easy to reason about mechanistically and intrinsically precise in where and when activity appears, although the decaging step is irreversible and relies on short wavelengths, with limitations in deep tissues 55.

Reversible protein switches are categorized into split reconstitution systems and single-chain photoswitches. In split systems (**Figure 3B**), Cas9 is divided into two inactive fragments. Their reassembly and subsequent activation are controlled by light, achieved by coupling the fragments to light-inducible dimerizers such as pMag and nMag 56. The activity rises with light and returns to baseline in the dark, which narrows the exposure window and facilitates dual-vector packaging for *in vivo* studies. Single-chain designs keep Cas9 intact but embed photoreceptor domains (pdDronpa1 or RsLOV) at strategic positions in the REC or PI regions so that the dark state sterically occludes DNA binding and illumination releases the active site, enabling reversible gating without selecting split sites (**Figure 3C, D**) 57 58. Both split and single-chain systems represent classic optogenetic strategies, in which genetically encoded photosensory modules convert optical signals into either protein-protein interactions or conformational unmasking, thereby enabling high spatiotemporal regulation of intracellular processes 59. These optogenetic switches have been applied to achieve subcellular-precision editing of endogenous genomic loci and to control complex biological events, such as precisely timing embryo implantation in mice, demonstrating their utility in dissecting dynamic gene functions in living systems 60.

Indirect optical control enables orthogonal regulation of Cas9 via light-gated inhibitors and expression systems. A key example is the LOV2-AcrIIA4 fusion, which acts as a photoswitchable brake: it suppresses the Cas9-sgRNA complex in darkness and releases inhibition under blue light to restore editing activity (**Figure 3E**) 61. This Acr logic has recently been broadened to AcrIIA5 and AcrVA1, enabling light and chemogenetic control across type II Cas9 and type V Cas12 orthologs with a unified, modular toolkit 62. An optochemogenetic strategy enables precise spatiotemporal and dosage control over Cas9. By fusing Cas9 to a destabilized DHFR domain, its stability becomes dependent on a photocaged trimethoprim (TMP) analog. Genome editing is thus activated only upon uncaging TMP with light, allowing independent control of dose (TMP concentration), timing (illumination), and spatial location (light targeting) at minute-scale resolution (**Figure 3F**) 63. At the expression layer, blue-light transcriptional switches such as GAVPO drive Cas9 expression from UAS promoters, which have supported targeted gene disruption of BRAF V600E in melanoma models and provide a convenient route when transient protein-level gating is not essential (**Figure 3G**) 64. Furthermore, a recent strategy installs a photocleavable group on the 5′ cap of Cas9 mRNA, blocking translation until illumination decages the cap, which complements protein and transcriptional switches by offering fast onset and low background (**Figure 3H**) 65.

**Figure 4:**
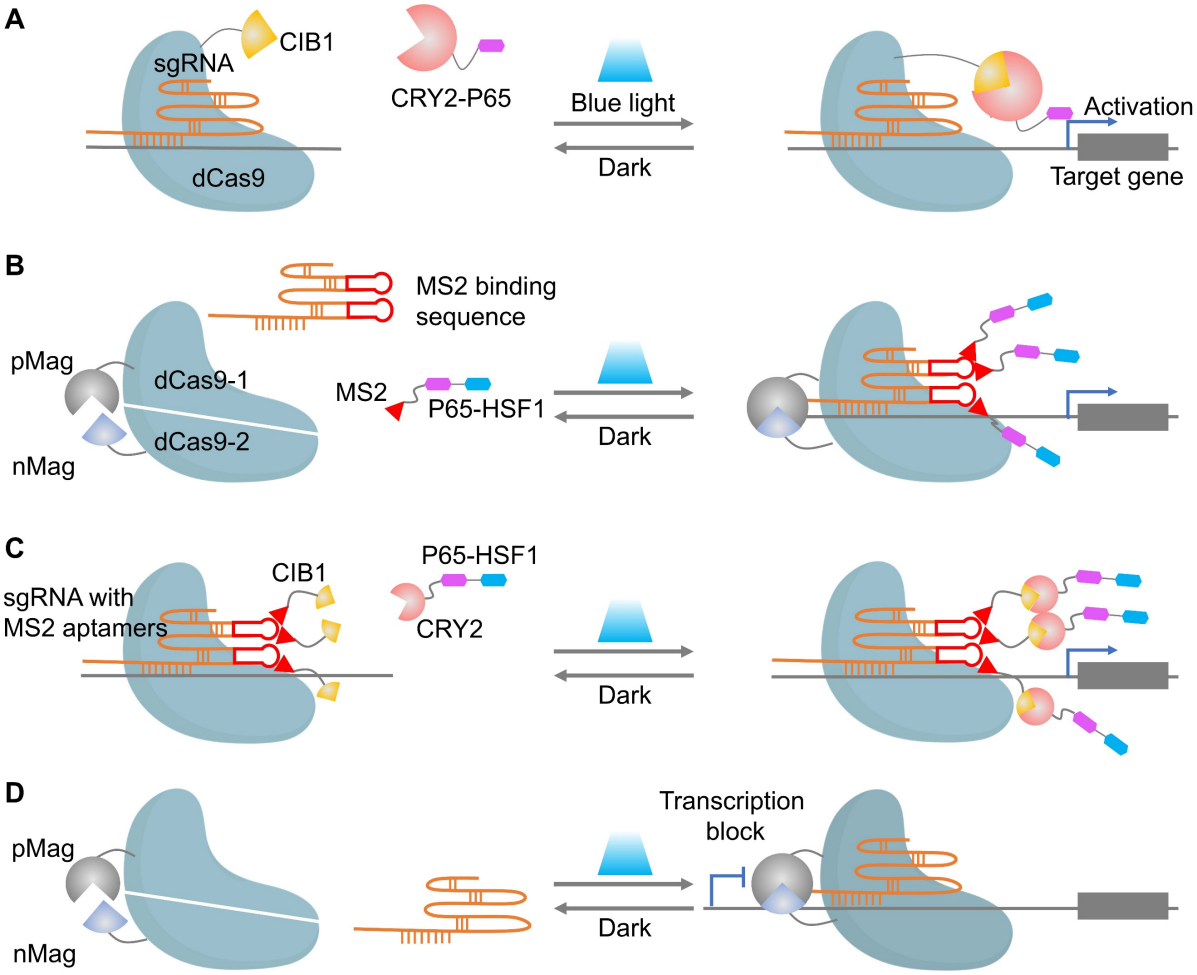
Representative cases of optical control of dCas9 function for target gene activation or inhibition. (A) A schematic of CPTS. (B) A schematic of split-CPTS2.0. (C) A schematic of CPTS2.0. (D) A schematic of padCas9.

Naturally, the above optical strategies can be partially extended to dCas9 transcriptional control and to nCas9-based base editors. Optical control of dCas9 has become a powerful tool for precise spatiotemporal regulation of gene expression. The core principle revolves around fusing dCas9 to light-sensitive proteins, allowing activation or inhibition of transcription upon light exposure (**Figure 4**). Early systems like CPTS and LACE relied on the blue-light-induced interaction between CIB1 and CRY2 to assemble functional dCas9 and drive gene activation (**Figure 4A**) 66, 67. However, these first-generation systems exhibited limited activation efficiency, which was addressed by Split-CPTS2.0 (**Figure 4B**), a refined version that enhances transcriptional output using VP64 and MS2 RNA aptamers 68. Despite its success, Split-CPTS2.0 suffers from slow deactivation kinetics, which led to the development of CPTS2.0 (**Figure 4C**), offering faster gene switching but lower activation strength compared to Split-CPTS2.0. These systems are complemented by photoactivatable CRISPR interference (CRISPRi) systems, such as padCas9 (**Figure 4D**), which allow for reversible transcriptional repression via light-induced dCas9 reassembly 56. Additionally, several innovations leverage light-induced protein interactions to regulate gene expression more dynamically 69-71, while LINuS uses light-induced nuclear localization to suppress gene activity effectively 72. Inspiringly, the combination of other repressors (such as KRAB) with optogenetic tools can generate more light-inducible gene suppression systems 73. These advances in light-controlled dCas9 technologies underscore their flexibility and power in both gene activation and repression, with substantial implications for functional genomics and therapeutic gene regulation.

**Figure 5:**
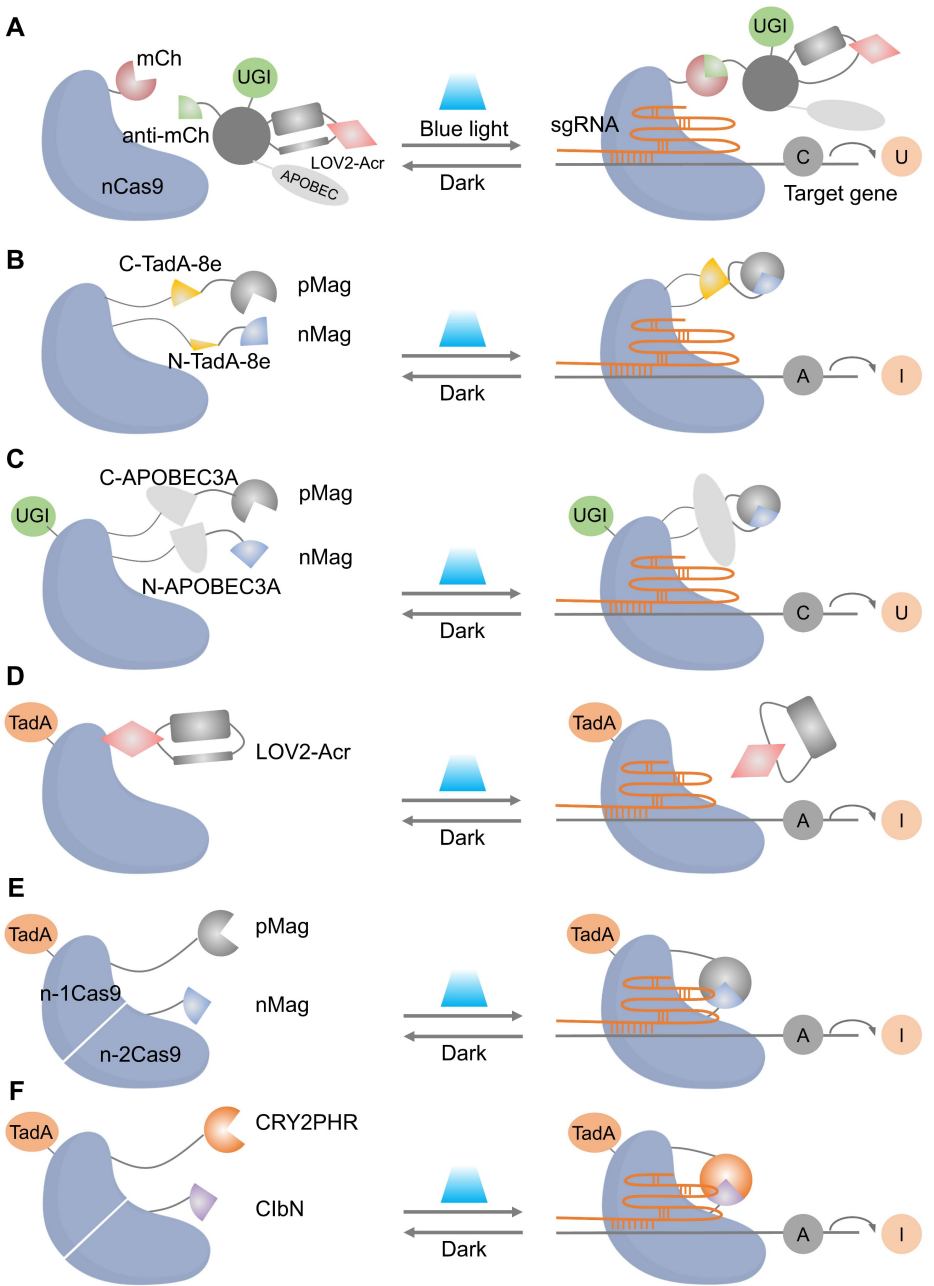
Representative cases of optical control of nCas9 function for target gene activation or inhibition. (A) A schematic of photoactivatable CBEs incorporating a sunbody. (B) Schematic of BLABE. (C) Schematic of BLCBE. (D) A schematic of CASANOVA-ABE system. (E) A schematic of Mag-ABE system. (F) A schematic of CRY-ABE system.

Light-gated base editing systems combine nCas9 with a light-controllable deaminase domain, enabling precise C-to-U or A-to-I conversions within a narrow editing window without generating DSB. This approach minimizes genotoxicity while allowing precise installation or correction of disease-related variants under tight spatiotemporal control. Representative strategies include photoactivatable CBEs (e.g., Sunbody-CBE, **Figure 5A**) 74, split-deaminase systems activated by blue light (BLABE/BLCBE, **Figure 5B-C**) 75, and ABE platforms (CASANOVA-ABE, CRY-ABE, Mag-ABE, **Figure 5D-F**). Notably, the Mag-ABE system has demonstrated *in vivo* efficacy in proof-of-concept studies 76. These systems collectively establish that reversible, on-demand base editing is achievable, offering a crucial advantage in scenarios where sustained deaminase activity poses risks.

Despite the above light-inducible methods having enabled dynamic regulation of endogenous genes with spatiotemporal precision, nearly all of them are activated by visible light (400-650 nm), particularly by blue light. The inherently shallow penetrative capacity of short-wavelength light severely limits these systems for *in vivo* applications through turbid tissues 77. To overcome these limitations, systems excited by far-red light (FRL, 650-750 nm) or near-infrared light (NIR, > 700 nm) with deep tissue penetration have increasingly attracted attention. Ye's group developed an optogenetic FRL-activated CRISPR-dCas9 Effector (FACE) system, which is orthogonal, fine-tunable, reversible, and deep tissue-penetrative for biocompatible, robust endogenous gene activation 40, 78. Furthermore, the following developed REDLIP provides second-scale ON/OFF switching and has been adapted to drive dCas9-based transcription (REDLIPcas) and therapeutic transgene control in mice, achieving robust *in vivo* performance under red/far-red illumination 79. In parallel, NIR actuation has been realized by engineering a photocleavable dimerization complex that unlocks split-Cas9 or split-dCas9 upon NIR exposure, delivering rapid, spatially precise activation while matching clinically favorable optical windows and being explicitly framed as compatible with human *in vivo* use 80. Complementary NIR routes pair editors with upconversion nanotransducers, which convert NIR to visible light to trigger established blue-light switches, such as Mag-ABE, which has been rendered NIR-addressable for liver base editing in reporter mice 76. Together, these FRL/NIR platforms extend optical control of (d/n)Cas9 into deep tissues while retaining fast kinetics and spatial addressability, and they provide practical scaffolds for translational studies *in vivo*.

Collectively, optical control of (d/n)Cas9 now encompasses irreversible decaging, reversible reconstitution or unmasking, inhibitor-based braking of the ribonucleoprotein, and expression-level regulation, with optogenetics providing the unifying paradigm. The field is steadily moving to FRL/NIR windows that suit tissue work while keeping fast kinetics and focal addressability. Continued attention to dark-state leak, illumination dose, and standardized on/off metrics will help translate these elegant molecular switches from cultured cells to deep-tissue settings and eventually to large-animal validation.

#### 2.1.2 Optical control of sgRNA function

Protein-centric optical systems have enabled precise (d/n)Cas9 control but may suffer from dark-state background (spontaneous assembly of split components) and prolonged illumination due to the limited photosensitivity of some dimerizers 56, 66. Alternatively, sgRNA, which plays a decisive role in Cas9 targeting, can also be engineered with photo-inducible strategies to enable the optical control of Cas9 function with spatiotemporal precision. Up to now, several chemical groups have been utilized for building photoactivatable sgRNAs to conditionally regulate CRISPR-Cas9 function. The related structures and properties are detailed in **Table 2**.

Most photo-ON designs work by masking the spacer so that Cas9-sgRNA pairing or target hybridization is blocked until light removes the mask, restoring activity with second-minute latency 91. The seminal CRISPR-plus approach used photocleavable ssDNA “protectors” (ortho-nitrobenzyl) to occlude the protospacer, and UV photolysis releases the protector and re-enables cutting, establishing the basic “block-then-unmask” logic 81. Subsequent nitrobenzyl chemistries (for example, NPOM 82-84, 92, DMNEC 85, and modified vitamin E 86) have enabled high-speed optochemical control. This concept underpins high-speed implementations such as vfCRISPR, which positions caging in the PAM-distal protospacer to achieve sub-micrometer, seconds-scale DSB induction with minimal leak 84.

Beyond backbone caging, azobenzene-mediated photoswitching introduces reversible control. A representative design, G-quadruplex gRNA (GqRNA), embeds dicationic azobenzene derivatives (AZD^++^) at the 5′ end so that the dark/visible-light isomerization modulates a structured blocker that prevents Cas9 engagement 93. Then illumination converts the switch and restores editing or CRISPRa, enabling spatiotemporal programming without protein engineering. Complementing this, covalently cyclized gRNAs connect two or three “buckles” with a light-cleavable linker to create a conformationally restricted circle that is nuclease-stable and functionally OFF until photo-opened, simplifying synthesis relative to site-dense caging while preserving sharp OFF to ON contrast 87.

Optical control can also terminate editing by switching guides ON to OFF 94-97. A recent visible-light CRISPR-OFF strategy installs a vinyl-ether handle into RNA that undergoes phenanthrenequinone-triggered photo-click inactivation under visible light, thereby reducing Cas9 off-targeting while avoiding UV and improving practicality for turbid tissues 90. These guide-layer methods have been synthesized and compared across multiple platforms in recent reviews 36, 91, 98, which emphasize their speed, low dark activity, and modularity across genome editing and regulation.

The sgRNA-centric photoregulation delivers fast kinetics, high on/off contrast, and no protein re-engineering, enabling multiplexed control of gene editing. Remaining bottlenecks include UV reliance for many cages, oligo synthesis complexity for long guides, and irreversibility in classic photo-ON designs. The emergence of azobenzene photoswitches, circular photo-openable gRNAs, and visible-light CRISPR-OFF provides concrete routes to reversibility, improved tissue compatibility, and cleaner termination of exposure, offering broader applicability and priorities for *in vivo* translation.

#### 2.1.3 Optical control of non-viral delivery systems

**Figure 6:**
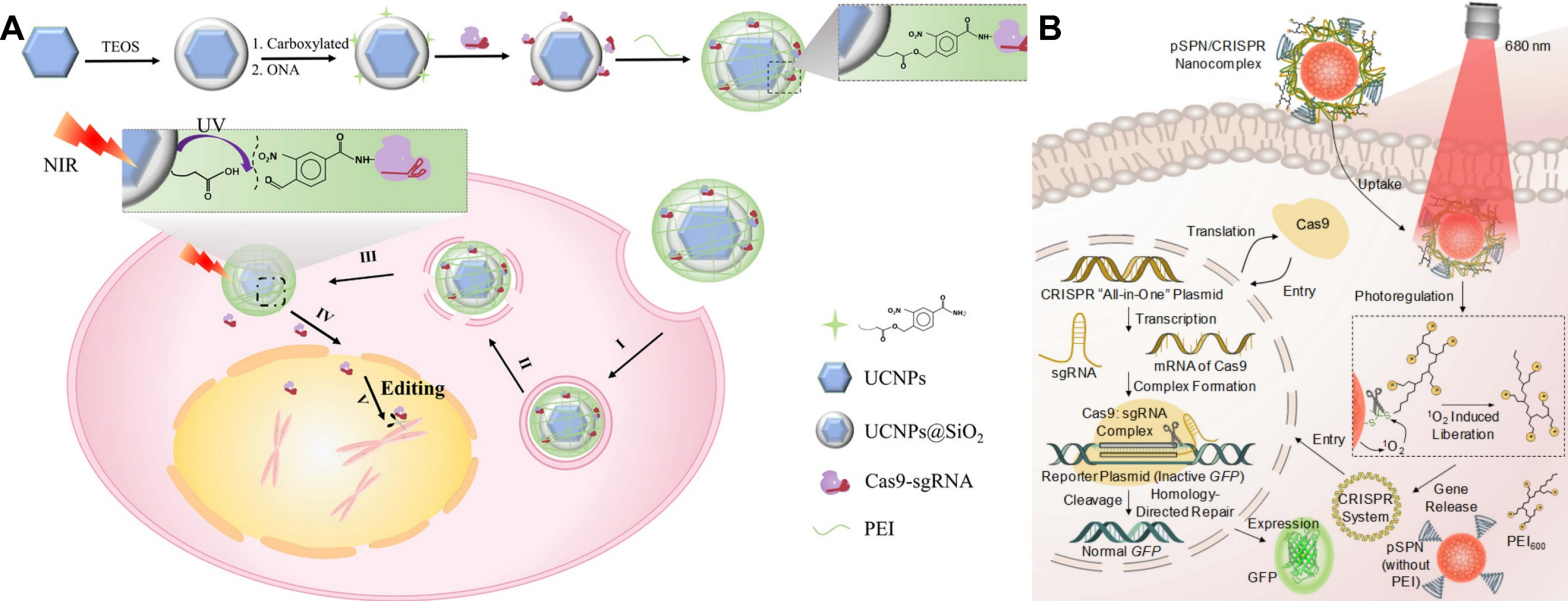
NIR light-controlled CRISPR-Cas9 delivery systems for gene editing *in vivo*. (A) Synthesis of UCNPs-Cas9@PEI and schematic illustration of NIR-triggered delivery of Cas9-sgRNA into the nucleus of a cell for gene editing. Reproduced with permission from 104, copyright 2019, AAAS. (B) Chemical structure of photolabile pSPN and schematic illustration of light-triggered delivery and release of CRISPR-Cas9 plasmids for gene editing. Reproduced with permission from 108, copyright 2019, Wiley-VCH.

Non-viral photoregulated carriers move the light gate from the editor to the vehicle, enabling on-demand release of CRISPR payloads with deep-tissue addressability and without re-engineering Cas9/sgRNA 99-101. One of the most mature routes is using lanthanide upconversion nanoparticles (UCNPs) for converting NIR excitation into local UV/visible light to cleave photolabile linkers or unlock nucleic-acid nanostructures 102, 103. Inspiringly, Song *et al*. designed an NIR light-responsive platform capable of spatiotemporal release of CRISPR-Cas9 complex (**Figure 6A**) 104. The CRISPR-Cas9 system was assembled with UCNPs through UV-photocleavable 4-(hydroxymethyl)-3-nitrobenzoic acid (ONA) molecules, and an outer polyethylenimine (PEI) coating was employed to enhance endosomal escape (UCNPs-Cas9@PEI). NIR irradiation generates short-wavelength emission at the nanoparticle, cuts the linker, and releases active CRISPR only at the illuminated site for tumor editing *in vivo*. Similarly, a charge-reversal UCNP vector (UCNP-UVP-P) uses NIR-induced UV to cleave a photosensitive linker and flip surface charge, boosting uptake and plasmid transfection of CRISPR-Cas9 with spatiotemporal control 105. The frontier is converging on programmable, multi-cargo NIR platforms, exemplified by UCNPs that execute spatiotemporally scheduled delivery of CRISPR-Cas9 106, 107. These UCNP strategies expand optical actuation from blue/UV to NIR, improving tissue reach while preserving focal addressability 107.

Another complementary class uses photolabile semiconducting polymer nanotransducers (pSPNs) (**Figure 6B**) 108. Here, the polymer backbone generates singlet oxygen under 680-808 nm light, which cleaves ^1^O₂-sensitive linkers that tether PEI brushes and electrostatically bound “all-in-one” CRISPR plasmids. Illumination thus triggers plasmid release and downstream editing in living mice. Unlike UCNPs, pSPNs do not rely on rare-earth dopants and can be tuned spectrally by polymer design, offering a chemically compact route to NIR-activated delivery.

Photoresponsive liposomes offer a clinically familiar chassis. By embedding verteporfin in the bilayer, 690 nm light generates singlet oxygen that transiently destabilizes the membrane and bursts Cas9 RNP payloads with single-cell spatial resolution (zebrafish), illustrating a regulatory path that leverages approved photosensitizers and established illumination hardware 109. In parallel, cell-membrane-coated nanoparticles extend circulation and homotypic targeting, highlighting their promise as stealthy CRISPR carriers and their compatibility with photo-triggered release modules 110.

Compared with previous protein- or guide-level photoswitches 50, 111, 112, optical non-viral delivery shifts induction into NIR windows to improve tissue penetration, confines editor exposure to the illuminated volume, and decouples molecular engineering from payload selection 104, 105, 108. But the key translational challenges remain, including systemic biodistribution and targeting, long-term nanomaterial fate, and rigorous characterization of light dose and thermal limits under clinical practice. Addressing these with targeting ligands, responsive shields, and calibrated NIR protocols will be key to advancing deep-tissue, image-guided genome editing.

### 2.2 Thermal control

Temperature substantially influences enzyme activity. For gene-editing nucleases, ZFNs and TALENs have been reported to show higher mutation frequencies under 30 °C in mammalian cells 113, 114. By contrast, SpCas9 activity is reduced under 30 °C but can be augmented under 39 °C, partly through increased enzyme kinetics and sgRNA expression, providing a lever to enhance on-target mutagenesis in eukaryotic cells 115, 116. Importantly, the temperature response is context dependent: In plants, transient heat stress from 22-28 °C to 30-37 °C reproducibly increases SpCas9 editing, whereas in mammalian cells maintained at 37 °C, hypothermia (e.g., 22-30 °C) impairs and mild hyperthermia (39 °C) can improve editing, underscoring the organism- and baseline-dependent nature of temperature effects 115, 116. These observations indicate that temperature is a tunable parameter rather than a universal optimum. Aside from the direct effect of temperature on Cas9 activity, thermal regulation of CRISPR-Cas9 can be achieved by other approaches, such as engineering temperature-sensitive Cas9 variants or inhibitors, incorporating heat-inducible promoters, and using heat-generating biomaterials.

#### 2.2.1 Temperature-sensitive Cas9 variants or inhibitors

Florian Richter *et al.* have screened and identified a temperature-sensitive RsLOV-Cas9 variant (tsRC9), in which the RsLOV domain, connected via a 21-residue C-terminal linker, is inserted between residues N235 and G236. The tsRC9 exhibits remarkable inhibition of RFP expression at 29 °C, while exhibiting minimal activity at 37 °C 58. As mentioned above, phages can utilize inhibitory “anti-CRISPR” (Acr) proteins to suppress CRISPR-Cas function in a sequence-independent manner. At least six inhibitors (AcrIIA1-AcrIIA6) have been identified to prevent DNA cleavage and genome editing by type II-A CRISPR-Cas9 enzymes 117. Interestingly, Jiang *et al.* discovered that AcrIIA2 displays potent temperature-dependent anti-CRISPR activity by blocking protein residues essential for DNA binding 118. What's more, AcrIIA2b shows lower temperature sensitivity and stronger inhibitory effect on Cas9 activity than AcrIIA2a at both room temperature (22 °C) and physiological temperature (37 °C), likely attributable to structural variations between the inhibitors and differences in their binding environments on Cas9. These results contribute to the development of a condition-dependent Cas9 toolbox for precise temporal and spatial genome editing.

#### 2.2.2 Heat-inducible promoter-mediated control

The heat shock transcription factors (HSFs) are a group of DNA-binding proteins involved in the transcriptional regulation of gene expression 119. Upon exposure to different stress factors, such as thermal stress and heavy metal exposure, HSFs undergo a conformational transition from inactive monomer to a DNA-binding-competent homotrimer, which subsequently translocates to the nucleus. These homotrimers then bind to the heat shock element (HSE) within the promoter region, thereby initiating transcription. Accordingly, HSE-containing promoters such as HSP70 have been used to build heat-inducible CRISPR-(d)Cas9 systems that enable spatially and temporally confined editing or activation with limited off-target effects 120-123. Notably, pulsed heating regimens have been shown to significantly enhance cellular thermal tolerance compared to continuous heating, thereby facilitating sustained gene regulation in cells. Considering that the optimal temperature for activating the HSP70 promoter ranges from 40 °C to 45 °C, precise temperature control is crucial to achieving a balance between activation efficiency and minimizing thermal damage. However, a recent tunable and reversible thermo-inducible bio-switch addresses these limitations by operating within physiological temperatures (approximately 26-37 °C), offering adjustable activation thresholds, low basal leak, rapid on-off cycling, and strong dynamic range 124. Because it functions without high thermal loads, this switch can be integrated with dCas9-based CRISPRa/CRISPRi or nuclease Cas9 in temperature-sensitive tissues, supports repeated activation schedules. In practical terms, it broadens thermal control from “heat-shock” to “physiological-range” regulation, reducing safety concerns while preserving spatiotemporal precision and thereby improving the translational outlook for temperature-gated genome engineering.

#### 2.2.3 Biomaterials-mediated thermal control

From the perspective of thermal regulation, a distinctive form of thermally responsive genome regulation, driven by heat generated from photothermal nanomaterials under light irradiation, has garnered significant attention 46, 120. Over the past decades, various photothermal nanomaterials have been developed, including noble metal nanomaterials, metallic compound nanocomposites, carbon-based nanomaterials, and organic semiconducting nanomaterials, demonstrating remarkable performance in drug delivery, oncotherapy, sterilization, and other biomedical applications 125, 126. It is highly appealing to achieve spatiotemporal control of CRISPR-Cas9-based genome engineering with photothermal nanomaterials under light irradiation. Accordingly, Jiang's group reported a photothermal-triggered CRISPR-Cas9 release system based on lipid-encapsulated gold nanoparticles (LACP) for tumor therapy 127. Upon 514 nm laser irradiation, the LACP system harnesses the photothermal effect of gold nanoparticles to release Cas9-sgRNA plasmids into the cytosol, enabling PLK-1 gene knockout and tumor suppression both *in vitro* and *in vivo*. The core gold nanoparticles functioned as a good photothermal bomb in the lysosome escape. The LACP system provides a flexible platform for efficient CRISPR-Cas9 delivery and precise gene editing, enabling the treatment of a broad range of diseases. Nevertheless, it is worth noting that short-wavelength light-triggered delivery systems may suffer from limited tissue penetration, restricting their applications to superficial tissues.

Compared to visible light (400-650 nm), NIR light possesses a longer wavelength and exhibits reduced tissue scattering and absorption in biological tissues, thereby minimizing photodamage and enabling deeper tissue penetration 128. Herein, Chen's group proposed a NIR-light-triggered remote control strategy for CRISPR-Cas9 genome editing, utilizing a carefully designed semiconducting polymer brush (SPPF) 129. SPPF functioned not only as a carrier for Cas9 delivery but also promoted endo-lysosomal escape and controlled payload release via photothermal conversion under 808 nm NIR irradiation. This enabled efficient, site-specific gene editing both *in vitro* and *in vivo* with minimal toxicity. Moreover, SPPF exhibited a unique second near-infrared window (NIR-II) emission under 808 nm NIR irradiation, enabling real-time *in vivo* monitoring of the genome-editing system's distribution and guiding laser irradiation. The first near-infrared window (NIR-I, 700-900 nm) imaging and NIR-II (1000-1700 nm) fluorescence imaging are ideal for *in vivo* bioimaging owing to their reduced photon absorption and scattering, minimal tissue autofluorescence, and enhanced spatial resolution at greater depths 128 130. Undoubtedly, real-time NIR-II imaging-guided light-responsive remote control significantly enhances the spatiotemporal precision of the CRISPR-Cas9 gene-editing process, thereby enabling safer genome editing *in vivo*.

**Figure 7:**
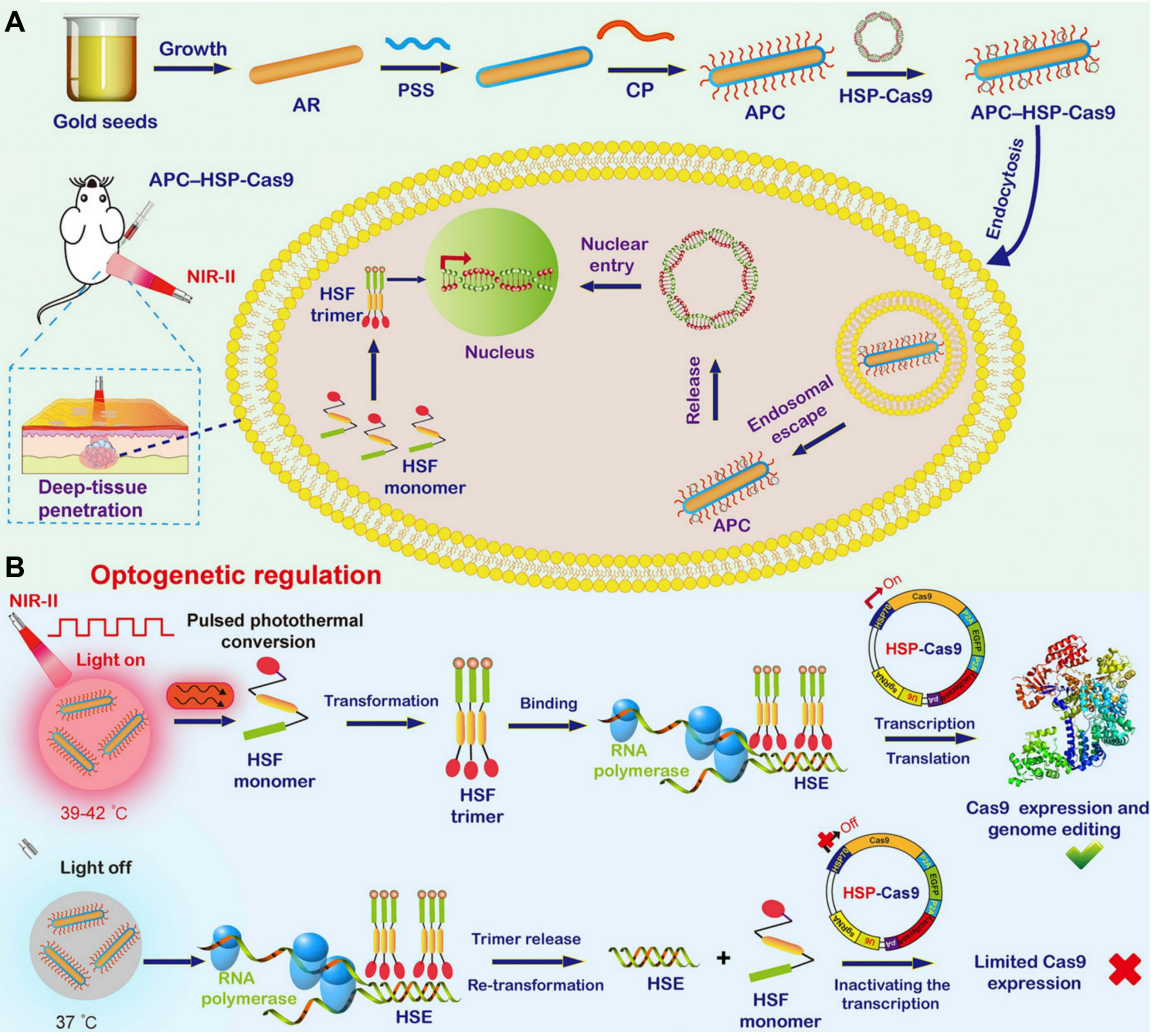
Illustration of the optogenetic regulation of genome editing mediated by the photoactivatable CRISPR-Cas9 nanosystem. (A) Synthesis of the nanoCRISPR system and illustration of intracellular delivery of HSP70-Cas9. (B) Illustration of optogenetic regulation of Cas9 expression for switchable genome editing under activation of NIR-II light. Reproduced with permission from 131, copyright 2020, PNAS.

Although various photothermal nanomaterials with gene delivery capabilities can be utilized for light-inducible Cas9 genome regulation with substantial or even superior efficacy, most of these light-triggered regulation processes are irreversible, and the activity of released Cas9 nuclease or sgRNA remains difficult to control reliably. Therefore, off-target activity induced by light-controlled genome editing remains elusive, highlighting the urgent need for precise CRISPR-Cas9 genome editing in an inducible, reversible, programmable manner. Herein, Ping's group reported an NIR-II light-activated optogenetic CRISPR-Cas9 nanosystem (nanoCRISPR) for programmable genome editing (**Figure 7**) 131. The nanoCRISPR system consisted of a gold nanorod functionalized with a cationic polymer and loaded with Cas9-encoding plasmids under the control of a heat-sensitive promoter (HSP70). The APC served not only as a carrier for efficient delivery and transfection of HSP70-Cas9 plasmid but also as a photothermal converter, transforming NIR-II light into localized heat to activate HSP70-driven Cas9 endonuclease expression, thereby enabling target gene editing. Notably, the genome-editing activity could be precisely modulated and easily programmed by fine-tuning exposure time and irradiation time *in vitro* and *in vivo*. The exceptional control precision of the nanoCRISPR system substantially reduced the off-target effects associated with CRISPR-Cas9 genome editing. Furthermore, the NIR-II optical properties of the nanoCRISPR system facilitated therapeutic genome editing in deep tissues, as evidenced by its successful application in treating deep-seated tumors and rescuing fulminant hepatic failure. Subsequently, Ping's group further optimized this NIR-II light-inducible CRISPR-Cas9 system and utilized it to reprogram the tumor microenvironment, thereby enhancing cancer therapy through targeted disruption of PD-L1 and mild-hyperthermia-activated immunogenic cell death 39.

The previously mentioned NIR-activatable CRISPR systems have been applied in peripheral tissues but face significant challenges in the brain, including skull penetration constraints and the need for precise spatial activation. Ferreira's group developed an NIR-responsive nanosystem for brain gene editing, in which sgRNA-Cas9 ribonucleoproteins are tethered to nanoparticles via complementary oligonucleotides 132. NIR irradiation induces local heating, triggering complex release and enabling precise spatial control, demonstrating effective gene editing both in single cells *in vitro* and in the brains of reporter mice *in vivo*.

Herein, thermal control offers tunable, non-invasive regulation of CRISPR activity with deep tissue penetration, particularly through NIR-responsive nanomaterials. However, challenges include precise thermal dosing to avoid cytotoxicity, potential off-target effects from prolonged Cas9 activity, and the limited reversibility of most current systems. Future efforts should focus on developing milder, reversible thermal switches and integrating real-time temperature feedback for enhanced safety in therapeutic applications.

### 2.3 Magnetic control

The magnetic field is regarded as one of the most effective triggering conditions for externally responsive regulation of biological processes. In contrast to chemical and optical signals, which suffer from limited spatial control and penetration, the magnetic field can deeply penetrate the whole body without tissue attenuation or adverse effect 133. Recent studies have demonstrated that magnetic nanomaterials can regulate molecular or cellular processes and deliver cargos (drug/gene) *in vivo*, either mechanically, thermally, or through magnetogenetics, in response to a magnetic field 48, 134-136. Therefore, the magnetic control of delivery and activity of the CRISPR-Cas9 system also garnered significant interest.

#### 2.3.1 Magnetic field-guided delivery

Bao and colleagues reported that CRISPR-Cas9-mediated genome editing can be spatially activated *in vivo* through a magnetic field, by combining magnetic nanoparticles with recombinant baculoviral vectors (MNP-BVs) 38. In this magnetic-responsive Cas9 delivery system, serum complement-inactivated baculoviral vectors function with an “off” switch effect to restrict their systemic activity, while an external magnetic field acts as an “on” switch, enabling tissue-specific genome editing by enhancing the margination and cellular uptake of MNP-BVs locally. The MNP-BVs facilitate *in vivo* liver-specific delivery, gene expression, and gene editing via systemic injection, guided by a magnetic field. In fact, achieving stringent spatial control over Cas9 expression or activity in the target organs and tissues *in vivo* remains challenging. Although optogenetic control of Cas9 offers high spatiotemporal precision, it still necessitates the use of invasive embedded fibers *in vivo*, owing to the limited penetration of optical light. In contrast, an external magnetic field can be easily applied with noninvasive manipulation and is not significantly attenuated by tissue. Additionally, due to the large packaging capacity of baculoviral vectors, the MNP-BVs-based delivery system holds promise for multiplexed genome editing *in vivo*.

In addition to targeting peripheral organs and tissues (such as liver and tumors), the magnetic-responsive nano-delivery system can cross the natural blood-brain barrier (BBB) for the treatment of brain diseases. Madhavan Nair's group demonstrated a magnetically guided, non-invasive platform employing magneto-electric nanoparticles (MENPs) for the targeted delivery and on-demand release of Cas9-gRNA across the blood-brain barrier to inhibit HIV 137. First, monodispersed ferromagnetic MENPs with positive surface charge electrostatically absorbed Cas9-gRNA and noninvasively navigated it across the BBB under the guidance of a static magnetic field. Then, an externally applied, optimized magnetic field generated by an electromagnetic coil enabled the controlled, on-demand release of Cas9-gRNA. This developed magnetically guided nano-formulation has paved a distinctive way for noninvasive CRISPR-based therapy of central nervous system diseases.

#### 2.3.2 Magnetothermally-triggered delivery

Magnetothermal heating is achieved through using an alternating magnetic field (AMF) to make magnetic nanoparticles flip or rotate their magnetic moments, generating local heat 138. Compared with the photothermal effect, magnetothermal control avoids optical scattering, enabling uniform heating with negligible tissue attenuation and no implanted optics, which is an attractive feature for *in vivo* CRISPR gating. Magnetothermally-triggered delivery systems have sparked a rapidly growing interest due to the ability to locate targets deeply inside a biosystem and potentially reduce off-target adverse effects 139. In a recent study, Li *et al.* engineered superparamagnetic core-shell ZnCoFe₂O₄@ZnMnFe₂O₄ nanoparticles coated with PEI and hyaluronic acid to electrostatically load CRISPR plasmids targeting HSP70 and BCL2 140. Under an alternating magnetic field, mild hyperthermia (~42 °C) accelerates plasmid release and transcription while simultaneously sensitizing tumor cells, thereby magnetothermally “switching on” dual-gene editing and amplifying apoptosis. In 4T1 models, the combined nanoparticle and magnetothermal regimen achieved significant tumor inhibition *in vivo* without overt systemic toxicity. Conceptually, this work couples deep-tissue, wirelessly addressable heat with CRISPR-Cas9 to overcome the penetration and phototoxicity limits of light, and it demonstrates a practical route to temperature-gated editing that synergizes with apoptosis pathways rather than relying on ablative temperatures.

#### 2.3.3 Magnetogenetics-based control

Magnetogenetics is an emerging neuromodulation and cell-control modality that couples time-varying magnetic fields to genetically sensitized cells through magnetic nanoparticles 53. The field converts magnetic energy into mechanical torque at the membrane, opening mechanosensitive channels (such as Piezo1) and translating a physical input into calcium signaling with millisecond-to-second resolution. Unlike optics, magnetic fields penetrate tissues with negligible attenuation, which enables fully wireless control *in vivo*. In the recent work from Lee's group 141, magnetomechanical forces from magnetic nanostructures activate Piezo1 on engineered target cells, trigger Ca²⁺ influx, and drive a Ca²⁺-responsive transcriptional program that expresses Cas9, thereby enabling remote, target-specific CRISPR editing. Compared with magnetothermal approaches, this mechanomechanical route avoids bulk heating and phototoxic concerns, and leverages endogenous Piezo1 signaling for rapid ON-OFF control governed by ion-channel gating kinetics. It thus offers a complementary magnetic control axis for CRISPR that is intrinsically compatible with deep tissues and can be tuned by nanoparticle design and field parameters.

**Figure 8:**
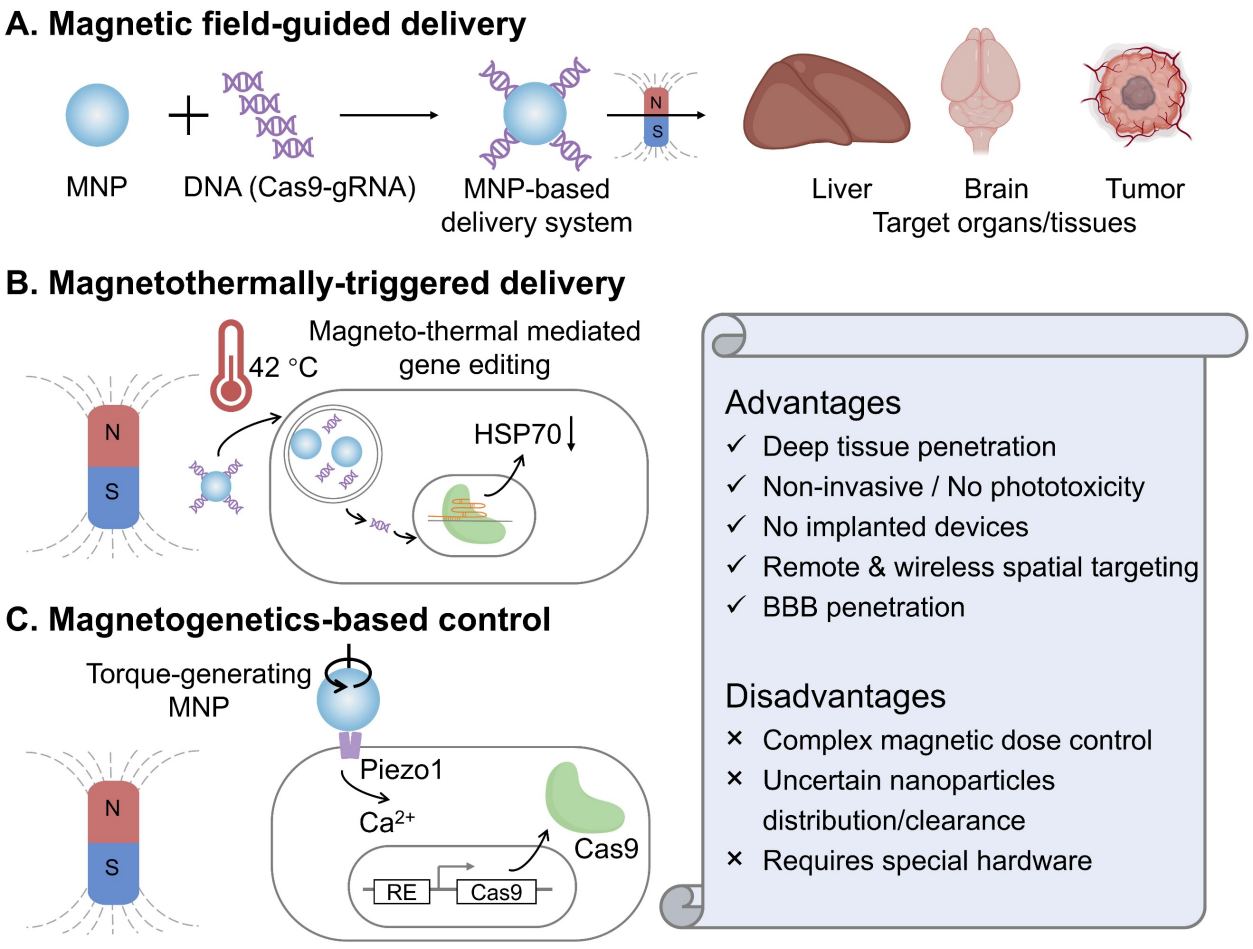
Magnetic control strategies for CRISPR-Cas9 systems and their key characteristics. (A) Magnetic field-guided delivery employs magnetic nanoparticles (MNPs) to complex with CRISPR-Cas9 components, enabling targeted accumulation at specific sites via an external magnetic field. (B) Magnetothermally-triggered delivery utilizes MNPs that generate heat under an alternating magnetic field, triggering the release of CRISPR-Cas9 cargo from thermally responsive carriers or inducing heat shock protein (HSP) promoters to activate gene editing. (C) Magnetogenetics-based control leverages torque-generating MNPs to mechanically stimulate specific ion channels (e.g., Piezo1), thereby activating downstream calcium (Ca²⁺) signaling and Cas9 expression. The lower panel summarizes the general advantages and disadvantages of magnetic control modalities.

The above exploratory studies indicate that magnetic control can enable deep and wireless stimulation of CRISPR-Cas9, especially when coupled with magnetothermal and magnetogenetic mechanisms (**Figure 8**). However, this field is still in its infancy, and many important questions remain unsolved, such as accurate dosimetry within clinically acceptable amplitude and frequency ranges, quantitative mapping of nanoparticle distribution, retention, and clearance *in vivo*, and reliable targeting and field focusing to avoid unintended heating or mechanostimulation.

### 2.4 Acoustic control

Ultrasound is one of the most widely used external stimuli in disease theranostics due to its ease of use, non-invasive nature, and ability to penetrate deep tissues 142. Originally, methods that generate pores in cell membranes through physical deformation, such as microinjection, electroporation, optoporation, microfluidics with constriction, and sonoporation, are commonly used to deliver desired biomolecules 143-147. Building on these foundations, emerging studies now harness ultrasound-responsive carriers to deliver CRISPR-Cas9 and leverage sonogenetic control to regulate CRISPR-Cas9 activity, drawing substantial interest across basic and translational research.

#### 2.4.1 Ultrasound-responsive delivery

First, a noninvasive, high-frequency ultrasound technique, termed acoustic-transfection, was established for remote intracellular delivery of CRISPR-Cas9 plasmids, enabling precise spatiotemporal monitoring and analysis of gene editing at the single-cell level 148. Unlike conventional sonoporation, acoustic-transfection employing ultra-high frequency ultrasound (>150 MHz) enables direct cytoplasmic delivery of genes and proteins without the use of microbubbles, thereby minimizing cavitation-related genomic damage. Nevertheless, further improvements are needed to achieve highly effective and efficient intracellular delivery of the Cas9-sgRNA complex. Herein, an ultrasound-powered nanomotor-based approach for the rapid, direct intracellular delivery of a functional Cas9-sgRNA complex was reported 37. The Cas9-sgRNA complex is anchored to thiol-functionalized gold nanowires (AuNWs) via reversible disulfide bonds. Upon ultrasound stimulation, the active and rapid movement of these Cas9-sgRNA/AuNWs enables their direct, rapid internalization into the cytoplasm. Subsequently, the Cas9-sgRNA complex was autonomously activated by the high intracellular concentration of glutathione within tumor cells, enabling highly effective GFP-gene knockout. This nanomotor-driven, ultrasound-guided strategy offers a compelling means for the direct and effective cytoplasmic delivery of functional Cas9-sgRNA complexes, highlighting its substantial potential for high-efficiency therapeutic gene editing.

Besides applications at the cellular level, acoustic control of CRISPR-Cas9 has also been tested in living animals. For example, Jee-Yeon Ryu *et al*. designed a microbubble-nanoliposomal carrier to effectively deliver and release Cas9-sgRNA complex at a specific site under local ultrasound activation 149. The Cas9-sgRNA complex effectively recognized and edited the target gene with high efficiency both *in vitro* and *in vivo*, demonstrating promising performance for androgenic alopecia therapy. Nonetheless, the low lysosomal escape rate during nonviral vector-mediated delivery of the CRISPR-Cas9 system remains a challenge for safe and effective gene editing *in vivo*. To address this, Xu's group engineered an ultrasound-triggered CRISPR-Cas9 delivery system (HMME@Lip-Cas9) for precise NFE2L2 knockdown, thereby mitigating adverse effects and improving the therapeutic efficacy of sonodynamic therapy (SDT) 150. Under ultrasound stimulation, this system can generate abundant reactive oxygen species (ROS) to kill tumor cells, as well as induce lysosomal rupture to release Cas9-sgRNA complex and destroy the oxidative stress defense system, achieving high-performance tumor therapy. In comparison to light-triggered CRISPR-Cas9-mediated gene editing systems, SDT offers significant advantages, including deeper tissue penetration and enhanced lysosomal escape ability, providing a new approach for establishing controllable CRISPR-Cas9 delivery systems with promising clinical translation.

In recent years, integrating nanotechnology with microbial carriers has emerged as a promising strategy to overcome physiological barriers such as tissue penetration, tumor hypoxia, and the blood-brain barrier 151. Xu's group developed a system (LGG-MHS) by combining hypoxia-targeting *Lactobacillus rhamnosus* GG with an ultrasound-responsive CRISPR/Cas9 platform 152. Upon ultrasound activation, the system generates ROS, inducing tumor-associated antigen release, immunogenic cell death, and dendritic cell maturation. ROS also facilitates endosomal/lysosomal escape of Cas9/sgRNA, enabling efficient nuclear translocation and IDO1 knockdown, thereby reducing Treg accumulation in the tumor microenvironment. This work underscores the potential of microbial vectors for *in vivo* CRISPR/Cas9 delivery, advancing clinical gene-editing applications.

#### 2.4.2 Sonogenetics-mediated control

Like optogenetics and magnetogenetics, sonogenetics uses focused ultrasound to modulate cellular functions in a remote and reversible manner through genetically encoded or engineered acoustic responsiveness 153. It offers deep penetration, fine spatial targeting, and noninvasive repeatability, making it readily compatible with imaging-guided interventions.

Two recent studies exemplify how sonogenetics can regulate CRISPR-Cas systems *in vivo*. Wu *et al.* engineer heat-inducible gene circuits so that brief, mild focused-ultrasound hyperthermia (~42-43 °C) induces CRISPR-dCas9 gene activation (CRISPRa), and an epigenetic editor (CRISPRee). They demonstrate precise, on-demand genome and epigenome modulation with spatial confinement and programmability suitable for immuno-oncology applications 54. Chen *et al.* design a thermosensitive genome-editing nanodevice that converts ultrasound-generated mild heating into intratumoral Cas9 activation. By editing tumor survival pathways and remodeling the microenvironment, this strategy enhances adoptive T-cell therapy efficacy while minimizing off-target exposure through localization of the trigger 154. These studies establish a generalizable framework in which focused ultrasound delivers localized thermal energy to actuate CRISPR machinery exclusively at the insonated site. This approach enables noninvasive, deep-tissue, and repeatable control over genome editing, transcription, or epigenetics. By combining established ultrasound hardware with modular CRISPR tools, it offers precise spatiotemporal gating and potential for imaging-guided operation. Key future priorities include standardizing thermal dosimetry, ensuring biosafety under repeated exposure, and scaling the technology for human applications. If successfully developed, sonogenetic control could become a practical clinical switch for spatially and temporally precise CRISPR therapeutics.

### 2.5 Other physical stimuli-responsive CRISPR systems

As previously mentioned, there are currently nearly 40 subtypes of CRISPR systems to date, each with a distinct pathway to achieving DNA or RNA editing. Developing stimuli-responsive control platforms of other CRISPR systems is also highly appealing for expanding the toolkit for mammalian genome engineering with spatiotemporal precision. Here, we focus on two well-studied examples: (d)Cas12 and (d)Cas13.

#### 2.5.1 CRISPR-(d)Cas12

**Figure 9:**
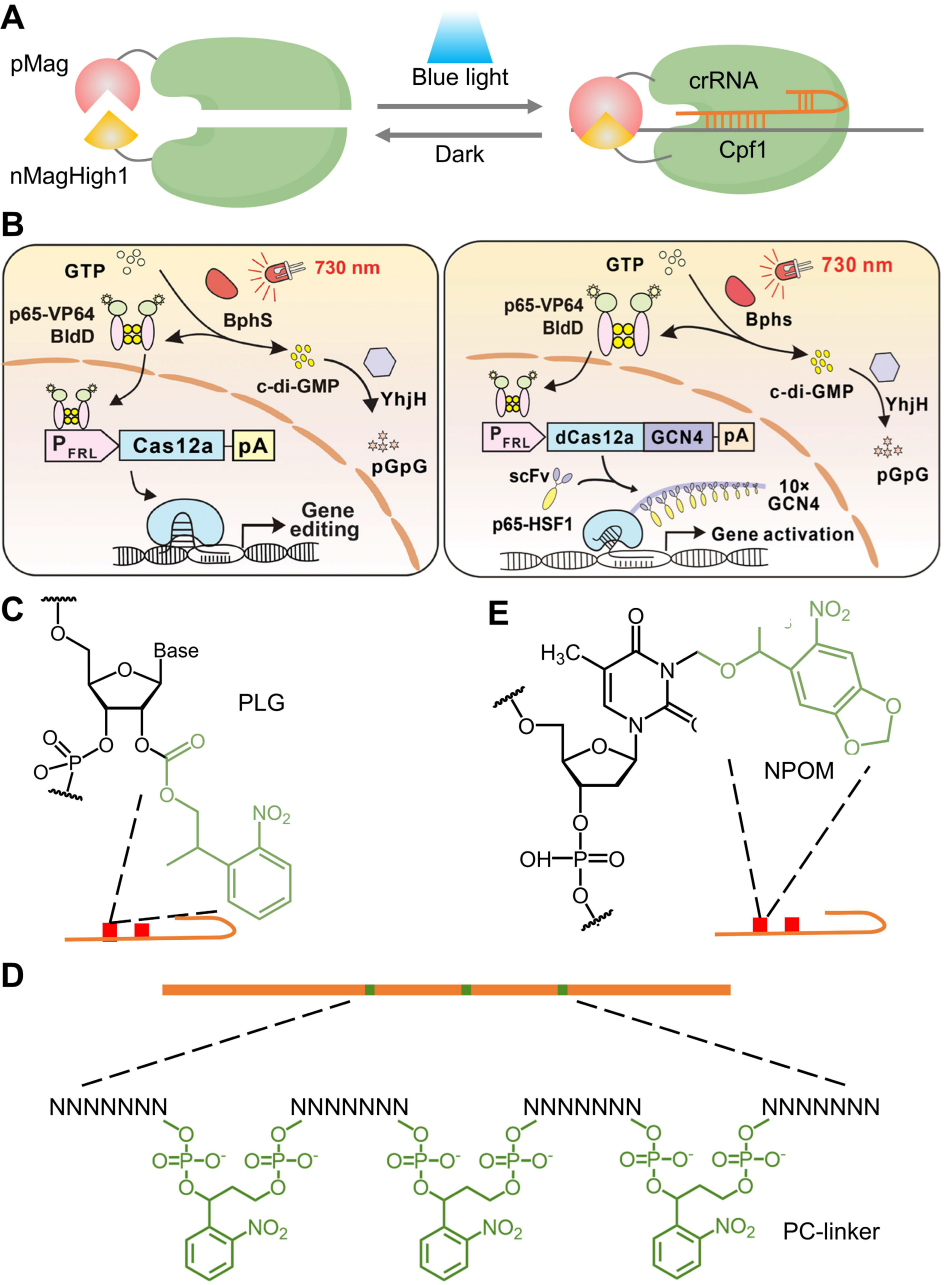
Physically tunable CRISPR (d)Cas12a systems. (A) A schematic of the light-inducible split Cpf1 platform. (B) Schematic design of the FICA and FIdCA system for gene editing and gene activation, respectively. Reproduced with permission from 158, copyright 2021, AAAS. (C) A schematic of ortho-nitrobenzyl phosphate ester-caged RNA hairpin structure. (D) Structural diagram of protective oligonucleotides. Protective oligonucleotides are linked by a spaced PC linker. (E) A schematic of NPOM-dt caged crRNA.

Recently developed CRISPR-Cpf1, also referred to as Cas12a, is another RNA-guided DNA nuclease with distinct properties, such as recognition of T-rich PAM, shorter crRNA, multiplex gene perturbation, and greater sequence specificity compared to Cas9 155-157. However, the improvement of the utility and versatility of Cpf1 requires significant improvement when compared with Cas9, which has a broader range of applications. Herein, Moritoshi Sato's group engineered a blue light-inducible split Cpf1 platform using the light-inducible dimerization domains (pMag and nMagHigh1), similar to the paCas9 system that was developed in their previous work (**Figure 9A**). This blue light-inducible Cpf1 system enables spatial, temporal, and reversible control of genome editing in mammalian cells without significantly higher off-target gene editing activity compared to that induced by the full-length Cpf1. However, the use of blue light as an illumination source has phototoxic effects on mammalian cells and limited tissue-penetrative capability *in vivo*. To address these challenges, Ye's group developed an FRL-inducible CRISPR-Cas12a (FICA) system and an FRL-inducible CRISPR-dCas12a (FIdCA) system (**Figure 9B**), utilizing the bacterial phytochrome BphS optical controllable system previously established by his group 158. Moreover, the FICA and FIdCA systems demonstrated greater efficiency in gene editing and gene activation when compared with the FAST and FACE systems developed in their previous works 40, 78, respectively.

The 2'-hydroxyl (2'-OH) is a characteristic functional group in RNA ribose, regardless of sequence, and reactions involving the 2'-OH group are, in principle, the most generalized pattern for covalent modification of RNA 159. Yang's group proposed a universal and accessible acylation strategy to regulate the CRISPR-Cas12a system by efficient acylation of 2'-hydroxyls (2'-OH) on the crRNA strand with photolabile agents (PLGs) (**Figure 9C**) 160. Zhou's group introduced an ortho-nitrobenzyl phosphate ester-caged RNA hairpin structure to control crRNA activation in CRISPR detection (**Figure 9D**) 161. The CRISPR-Cas12a activity is blocked by a PC linker containing a protective nucleic acid strand to temporarily silence the function of crRNA. The photocontrolled CRISPR assay separated the nucleic acid amplification and CRISPR reactions in the time dimension, realizing a closed, single-tube, high-sensitivity nucleic acid assay. However, the current method requires careful optimization of the ratio of PC-linker-containing oligonucleotides to guide RNA to achieve complete hybridization and silencing. In addition, a hybridization-based crRNA silencing strategy will prevent the prior binding of crRNA to Cas12a protein, thus affecting the Cas12a/crRNA stability. Hence, they developed a new light-start CRISPR-Cas12 system using photochemically activated caged crRNA (embed 6-nitropiperonyloxymethyl-caged thymidine (NPOM-dt) into the crRNA), which is simpler, faster, more stable, and more cost-effective (**Figure 9E**) 162.

In addition to direct regulation of the CRISPR-Cas12 system, light-responsive carriers have also been explored. Lei's group introduced a logic gate-based delivery system that spatiotemporally controls Cas12a RNP complexes using DNA-functionalized Mxenes 163. The release of these complexes was triggered by NIR-induced photothermal effects and nucleic acid strand displacement, enabling targeted gene editing. This method combines both external and internal triggers, offering a refined strategy for *in vivo* genome editing in therapeutic applications. These studies not only enhanced the utility and versatility of CRISPR-Cas12a (Cpf1) but also broadened the toolkit of precise gene engineering.

#### 2.5.2 CRISPR-(d)Cas13

**Figure 10:**
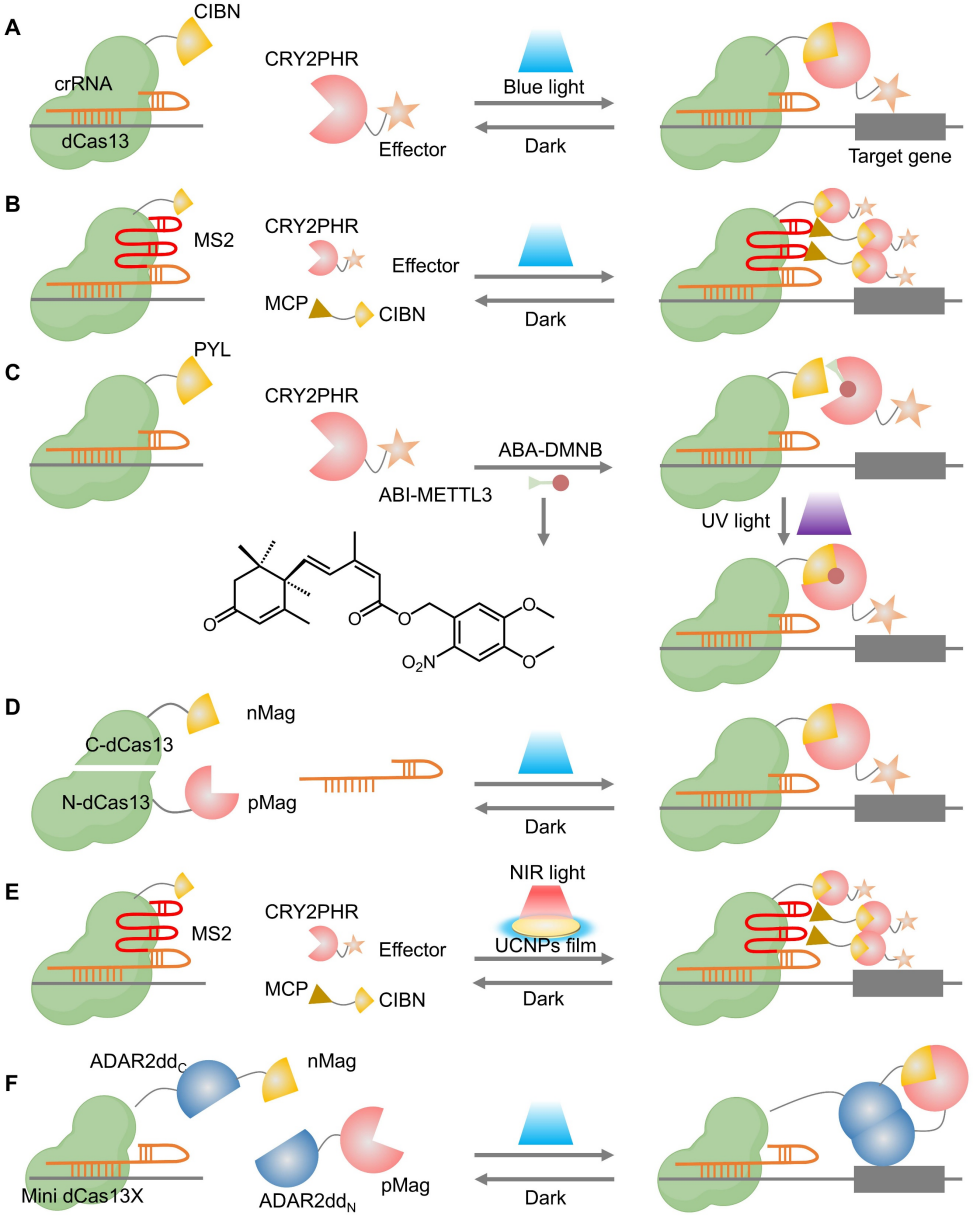
Physically tunable CRISPR (d)Cas13 systems. (A) A schematic of the PAMEC system. (B) A schematic of the PAMEC^R^ system. (C) The light-inducible m6A writing system incorporates photo-caged ABA-DMNB that can only be activated by UV light to regenerate functional ABA, which recruits ABI-M3 to enable m6A writing. (D) Schematic diagram of the photoactivatable Cas13 (paCas13). (E) A schematic of NIR light (980 nm)-activated PAMEC^R^ system with a UCNPs film. (F) Illustration of the RNA editing process facilitated by photoactivatable RNA base editors.

In addition to DNA, RNA plays a crucial role in the intricate and dynamic gene network. Consequently, the RNA-targeting CRISPR-(d)Cas13 platform has been developed for precise RNA editing, enabling the manipulation of biological processes at the RNA level 164, 165. However, robust and versatile tools for spatiotemporal precision in RNA manipulation remain limited. Herein, Zhao *et al.* developed a photoactivatable CRISPR-dCas13-based RNA N6-methyladenosine (m^6^A) editing system (PAMEC) and further optimized (PAMEC^R^) for specific m^6^A editing using the light-inducible heterodimerizing proteins (CIBN and CRY2PHR) (**Figure 10A-B**) 166. The PAMEC and PAMEC^R^ enabled efficient spatiotemporal control of m^6^A editing and allowed robust, simultaneous manipulation of multiple genes in response to blue light, respectively. To achieve precise and reversible control over RNA modifications, recent studies have integrated photo-regulation with chemical induction, offering a dual-responsive approach for epitranscriptomic editing. For instance, Shi *et al.* synthesized a UV light-controlled m6A editing platform, named abscisic acid (ABA)-4,5-dimethoxy-2-nitrobenzyl (DMNB), which offers enhanced spatiotemporal control compared to using a chemical ligand alone and can potentially allow for dosage-dependent regulation of effects, a challenge for light-inducible dimerization proteins (**Figure 10C**) 167. Similarly, Xie *et al.* constructed an ABA-inducible and photoactivatable m^1^A editing system (termed AI-dm^1^A) to achieve light-inducible m^1^A methylation or demethylation on specific transcripts 168. However, these inducers depend on chemical treatments, necessitating additional and complex procedures for *in vivo* use. Yu *et al.* presented a simple blue light-inducible method for RNA modulation by integrating a chemogenomic split-Cas13 system based on FKBP-FRB dimerization domains and an optogenetic photoactivatable Cas13 (paCas13) using the Magnet system (**Figure 10D**) 169.

Considering the limited tissue penetration of blue light and UV light, Zhao *et al.* further employed a UCNPs film as a light transducer to extend the range of activating light from the visible region to the NIR wavelengths that can penetrate tissue (**Figure 10E**), enabling remote control of the RNA editing. The Cas13-based optogenetic platforms for targeted, flexible, and efficient RNA engineering demonstrate broad applicability in epitranscriptome engineering, imaging, and future therapeutic development.

With the growing utility of optogenetic tools in RNA modification, recent efforts have extended their application to single-nucleotide RNA editing for enhanced precision in functional studies and therapeutic development. A photoactivatable A-to-I RNA base editor (PA-rABE) was developed, comprising a mini dCas13X and a split ADAR2dd fused to the Magnet system, which reconstitutes an active deaminase upon blue light exposure (**Figure 10F**) 170. This system enables efficient and programmable editing of both exogenous and endogenous transcripts *in vitro* and *in vivo*. PA-rABE offers spatiotemporally precise, single-base RNA editing with high specificity and minimal off-target effects, representing a powerful tool for transcriptome engineering.

In general, the development of other physically tunable CRISPR systems is still in the infancy stage, and more sophisticated inducible CRISPR systems with admirable efficiency and spatiotemporal precision are expected to be proposed for the research of complex gene networks.

## 3. Comparisons among different physical trigger-induced strategies

Overall, all four physical triggers (light, heat, magnetic, and ultrasound) can remotely activate the CRISPR-Cas9 systems for spatiotemporal genome engineering. However, each control modality has its own characteristics (**Table 3**). Comparisons among these physically inducible strategies in terms of spatiotemporal precision, tissue penetration, reversibility, off-target effects, biocompatibility, and respective limitations are further analyzed below.

### 3.1 Spatiotemporal precision

The CRISPR-Cas9 system with precise spatiotemporal control is essential for achieving desired genome engineering at a targeted site and specific time. Optical control generally delivers the highest spatiotemporal precision. By tuning wavelength, focus, power, and exposure, one can confine activation to subcellular and second scales, and switch on demand 66, 84. Thermal control inherits the precision of its energy source: with focused ultrasound heating, the “active” temperature window can be confined to a millimeter-scale focal zone 142. Ultrasound allows repeatable, 3D addressable focal volumes with good spatial confinement. Magnetic control depends on field gradients and on the ability of magnetic carriers to concentrate at the target. Its precision is typically organ- or lesion-level unless paired with image guidance and smart vectors 171.

### 3.2 Tissue penetration

Tissue penetration ultimately determines whether a controllable CRISPR-Cas9 system can be dosed at clinically relevant depths and volumes, making it an inevitable constraint for clinical translation 172. Optical approaches face wavelength-dependent limits. The ultraviolet/visible light typically reaches only hundreds of micrometers to a few millimeters in turbid tissue 50. Moving to the NIR I window (700-900 nm) extends practical penetration to the centimeter scale in favorable sites, while the NIR II window (1000-1700 nm) can further improve depth and signal-to-noise but requires specialized sources, optics, and materials 128. For deep targets, optical dosing often relies on fibers or implantable emitters 158. Ultrasound, by contrast, traverses centimeters to tens of centimeters (with deeper reach at lower frequencies) and can be tightly focused under image guidance, though bone and gas interfaces may distort beams 173. Magnetic fields themselves penetrate tissue with negligible attenuation, but focalization depends on achievable gradients and the *in vivo* behavior of magnetic carriers 135. Thermal control inherits the penetration of its energy source 142: focused-ultrasound heating offers deep, millimeter-scale foci, whereas photothermal schemes are bounded by the same optical limits described above.

### 3.3 Reversibility

Rapid reversibility could allow CRISPR-Cas9 activity to decrease or return to pre-stimulus levels upon removal of physical triggers or be stimulated by another inducer, thereby mitigating the adverse effects caused by prolonged CRISPR-Cas9 activity. Among the four physical control modes, optogenetic switches built on reversible dimerization or photoswitchable parts show fast, repeatable on/off behavior, often within minutes of light withdrawal 111. Thermal and some acoustic schemes can be reversible, but cooling and heat diffusion elongate the off-kinetics 123. Magnetic and acoustic modalities often need circuit-level designs (e.g., heat- or mechano-responsive promoters) to achieve rapid, repeatable reset 54, 141.

### 3.4 Off-target effect

Across physical modalities, gating reduces exposure-driven risks by confining where and when Cas9 is active. Optical control offers the tightest spatial confinement and rapid shutoff, which limits spatial off-targeting and temporal overexposure in accessible sites 111. Thermal control can confine activation to millimeter-scale foci with focused ultrasound heating, yet cooling extends the off-tail, so careful dosimetry is needed 122, 123. Magnetic control improves organ or region-level targeting through field-guided enrichment, but precision is limited by achievable gradients and vector kinetics, leaving some background exposure 171. Ultrasound reaches deep tissues with image-guided foci and repeatable pulses, though skull or air interfaces and carrier requirements may divert dose 142. However, previous studies have paid less attention to the “intrinsic off-target effect”, which largely depends on guide choice, nuclease fidelity, and cumulative on-time. Yu's work assessed the off-target effects of the FAST system by evaluating a single potential off-target site in the human BMP1 locus, with no mutations detected at this site after FAST system editing 40. Chen *et. al*. utilized an off-target search tool, Cas-OFFinder, to investigate the photothermal nanoCRISPR system, and demonstrated that this near-infrared optogenetics engineering system can minimize off-target effects by reducing prolonged Cas9 activity 131. Collectively, significant attention has been devoted to enhancing spatiotemporal control of physical stimuli-responsive CRISPR-Cas9 systems with reduced “exposure-related off-target effect”, while the optimal approach to minimize the “intrinsic off-target effect” of the CRISPR-Cas9 system remains unclear, requiring further investigation.

### 3.5 Biocompatibility

Physical stimuli-responsive CRISPR-Cas9 systems with good biocompatibility, including minimal invasiveness and negligible toxicity, are essential both for phenotypic studies after genome engineering and for clinical translation. For optical control, ultraviolet and visible light penetrate poorly in turbid tissue, often necessitating fiber implantation for deep targets *in vivo* 49. Phototoxicity further limits broad use, especially under prolonged illumination 52. Moving to near-infrared wavelengths improves safety and depth and is a practical route to deep-tissue gene editing when paired with careful dosimetry 102. For thermal, magnetic, and acoustic control, the external stimulus is noninvasive and broadly well tolerated, which largely circumvents the penetration limits of visible light. In most implementations, however, these modalities rely on responsive biomaterials such as photothermal, magnetic, or ultrasound-sensitive nanoparticles and polymers 37, 38, 131, 150. Their use introduces minimal invasiveness through local or intravascular administration, and the material's intrinsic toxicity directly shapes overall biosafety. Despite the general feasibility of activable genome engineering with acceptable tolerability, standardized and in-depth assessments and judicious selection of biosafe materials remain necessary to ensure that the benefits of tunable editing outweigh potential risks. Several key biosafety issues should be taken into consideration. First, immunogenicity may arise from protein editors, nucleic acids, and carrier chemistries, including complement activation and cytokine release, which can alter pharmacokinetics and provoke adverse events 25. Second, heat-related injury can occur if local temperatures or exposure durations exceed safe windows, highlighting the need for real-time thermometry and conservative cumulative dose management during photothermal, magnetothermal, or focused-ultrasound heating 174. Third, the long-term fate of biomaterials requires evaluation of organ retention, macrophage sequestration, fibrosis, and clearance, with preference for biodegradable or renally excretable designs to minimize chronic burden 175. Finally, acoustic bioeffects such as inertial cavitation should be monitored and constrained within validated safety indices 176.

## 4. Conclusion and outlook

The CRISPR-Cas9 system, with unprecedented simplicity and flexibility, has profoundly transformed the field of genome engineering. To achieve high precision and facilitate clinical translation of gene editing, numerous strategies have been developed to regulate CRISPR-Cas9 activity, particularly to meet the demands for high spatiotemporal control, deep penetration, rapid reversibility, minimized off-target effect, and excellent biocompatibility. Diverse CRISPR-Cas9 systems activated by external physical stimuli (light, heat, magnetic, and ultrasound) demonstrate significant advantages in these critical aspects. Despite these remarkable achievements, *in vivo* applications and clinical translation of physical stimuli-responsive CRISPR-Cas9 systems remain in their infancy, and several key issues warrant further investigation.

Biomaterials-based delivery systems have emerged as pivotal enablers for achieving precise spatiotemporal control over CRISPR-Cas9 genome editing 177. These systems offer distinct advantages over viral vectors, including enhanced safety profiles and expanded cargo capacity. Furthermore, the integration of stimuli-responsive elements, activated by biological or physical cues such as enzymes, pH, light, or ultrasound, has significantly improved the precision of genomic interventions 178. Notably, biomaterials can overcome the inherent limitations of certain control mechanisms. For instance, advanced optical systems employing second NIR-II window irradiation have substantially ameliorated the challenge of limited tissue penetration in photo-regulated CRISPR-Cas9 applications 179, 180.

Looking forward, several strategic directions are critical to advancing this field. First, multi-stimuli combinatorial control represents a promising paradigm for enhancing specificity and programmability. Current physical induction methods often operate unidirectionally. Future systems integrating orthogonal inputs, such as magnetic targeting coupled with optogenetic activation, or UCNPs-mediated wavelength-selective triggering, could furnish multiplexed and high-fidelity regulation, bridging the gap between unidirectional and truly programmable operation 181-184.

Second, while the translational potential of physically controlled CRISPR-Cas9 systems is immense, a substantial majority of reported platforms remain validated primarily in cell lines or murine models. Bridging this bench-to-bedside divide necessitates addressing key challenges:

(1) Immunogenicity and biocompatibility: Non-viral carriers and nano-formulations show promise, but their immunogenicity, both acute and long-term, must be rigorously evaluated, particularly in human models 185. This includes assessing the potential for inflammatory responses, organ-specific accumulation, and off-target immune activation. Long-term biosafety concerns, such as chronic inflammation, fibrosis, and the risk of unwanted immune responses, must be addressed before clinical application 186.

(2) Scalability and manufacturing: Translating lab-scale synthesis of CRISPR delivery systems into Good Manufacturing Practice (GMP)-compliant production is crucial for clinical adoption 187. This includes optimizing formulation stability, yield consistency, and quality control throughout large-scale production. Furthermore, the manufacturing processes must meet the stringent regulations for biopharmaceuticals and gene therapies 188.

(3) Safety profiling: Physical stimuli such as ultrasound or photothermal activation can raise concerns regarding heat-induced tissue damage. Tissue heating, especially in deeper organs, requires precise dose calibration, real-time temperature monitoring, and adaptive control systems to avoid unintended thermal injury 189. Real-time monitoring tools, integrated with imaging systems, must be developed to ensure the safety and efficacy of physical stimuli during *in vivo* applications. Moreover, long-term studies on the fate of nanomaterials used in CRISPR delivery are essential to identify potential toxicity and organ accumulation issues, which could limit the therapeutic potential of these systems 190.

(4) Regulatory and ethical pathways: As these technologies near clinical trials, regulatory frameworks for gene-editing therapeutics must be clearly defined, especially those that combine devices and biologics (e.g., CRISPR-loaded nanoparticles and ultrasound platforms). Regulatory bodies like the FDA and EMA will need to adapt existing guidelines to address the unique challenges of genome editing, including off-target effects, durability of edits, and long-term safety 191. Ethical considerations are equally critical, especially as CRISPR technology progresses from somatic cell therapies to potentially germline editing 192. Societal dialogues on germline editing and the implications of widespread genome manipulation will be necessary to ensure informed consent, oversight, and regulation 193.

Among the various physical stimuli, ultrasound and magnetic activation currently show particularly strong translational promise. These modalities offer deep-tissue penetration, non-invasiveness, and compatibility with existing clinical imaging systems such as MRI and ultrasound, facilitating their integration into established therapeutic workflows. Magnetic systems also benefit from the ability to target specific tissues with high precision 171, while ultrasound can provide real-time feedback, offering unparalleled control over delivery and activation 194. These features make ultrasound and magnetic-triggered CRISPR systems highly adaptable for clinical use. However, further systematic studies are required to validate their long-term safety, optimize their dosing parameters, and standardize their operational protocols for human therapeutic applications.

Finally, the potential for prevalent artificial intelligence (AI)-driven optimization presents an exciting frontier in CRISPR technology. AI models can enhance guide RNA design, predict editing outcomes, and personalize stimulation parameters based on real-time feedback, ensuring spatial and temporal accuracy in genome editing 195. Machine learning algorithms can analyze large datasets from preclinical trials to predict the most effective CRISPR delivery methods, further improving efficiency and minimizing off-target effects 196. As these AI technologies mature, they could provide an automated framework for personalized gene editing therapies tailored to individual patient needs, thus enhancing both safety and efficacy.

In summary, the next chapter of physically controlled CRISPR-Cas9 systems will hinge on synergistic advances in materials design, multi-modal control, translational rigor, and intelligent automation. By confronting the current translational gaps and harnessing emerging computational tools, we can accelerate the development of safe, effective, and clinically viable genome-editing therapeutics.

## Figures and Tables

**Table 1:** Advantages and disadvantages of chemical- and physical-inducible CRISPR-Cas9 control (overview).

Approach	Advantages	Disadvantages	Ref.
Chemical-inducible systems	Mature toolkits for transcriptional and post-translational control (e.g., Tet, chemically induced proximity, Cre system, degrons, anti-CRISPR protein) Simple to implement; no external hardware required Systemic reach via pharmacokinetics; convenient oral/intravenous dosing Potential multiplexing with orthogonal ligands	Limited spatiotemporal precision; distribution dictated by pharmacokinetics/biodistribution Slower on/off at the transcriptional level Significant background activity Inducers may perturb endogenous pathways; drug-drug interactions and immune/toxicity risks Reversibility constrained by inducer clearance; prolonged exposure risks	5, 34, 35
Physical-inducible systems	High spatiotemporal precision; focal and programmable exposure windows Typically rapid and reversible (stimulus-gated on/off) Low dependence on pharmacokinetics; tunable by device/field parameters Deep-tissue options available (thermal, magnetic, ultrasound); optics for surface/medium depth Natural orthogonality via wavelength/frequency/field strength Enhanced target specificity and biological applicability	Requires external hardware or materials Modality-specific risks (phototoxicity, thermal dose, cavitation, nanoparticle retention) Access/geometry constraints in certain organs *In vivo* maturity is uneven across modalities; device/material regulatory alignment is required	36-40

**Table 2:** Chemical structures for light-responsive regulation of gRNA function.

Name	Structure	Light wavelength	Reversible	Editing efficiency	Ref.
2-nitrobenzyl linker	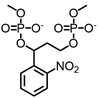	365 nm	No	Not mentioned	81
NPOM	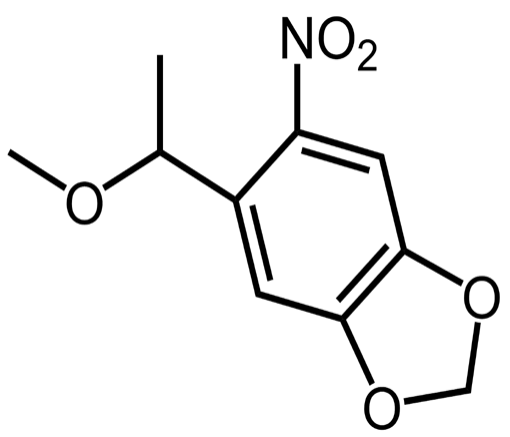	365 nm	No	Not mentioned	82-84
DMNEC	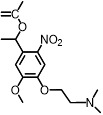	365 nm	No	Not mentioned	85
Modified vitamin E	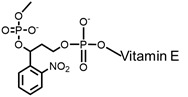	365 nm	No	> 50%	86
NPBOM	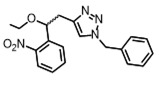	365 nm	No	> 40%	87
Coumarin amine	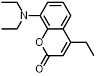	470 nm	No	> 20%	88
DEACM	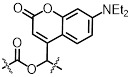	456 nm	No	Not mentioned	89
NB	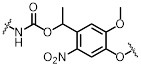	390 nm	No	Not mentioned	89
Vinyl ether	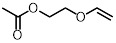	410 nm	No	< 5%	90

**Table 3:** Comparisons among different physical trigger-inducible CRISPR-Cas9 systems

Dimension	Optical	Thermal	Magnetic	Acoustic
Spatiotemporal precision	Highest; subcellular and second scales achievable	Depends on energy source; focused ultrasound can confine to mm-scale focal zone	Organ or lesion level; relies on field gradients and carrier enrichment	High; 3D addressable focal volumes at mm scale
Typical activation latency	ms-s (photoswitches or photocleavable caging)	s-min (heating to threshold and stabilization)	s-min (field-driven aggregation, heating, or circuit response)	s for mechanical, cavitation or thermal schemes
Reversibility	Best; minutes-scale recovery after light withdrawal, construct dependent	Moderate; limited by heat diffusion and cooling kinetics	Requires circuit-level design to enable rapid reset	Moderate; mechanical effects are fast, thermal routes limited by cooling
Tissue penetration	UV/Visible: <1-2 mm; NIR-I: up to ~1-2 cm in favorable tissues; NIR-II: deeper but requires specialized sources and materials; deep sites often need fibers or implants	Focused ultrasound -heating: centimeters; photothermal: limited by optical penetration as at left	Magnetic fields experience negligible attenuation; focalization is limited by achievable gradients and coil geometry	Centimeters to tens of centimeters; deeper at lower frequency; bone and gas interfaces may distort beams
Biomaterials required	Partial (photoswitchable proteins, photocages, photothermal or upconversion materials)	Partial (photothermal or magnetothermal materials; Focused ultrasound can reduce material needs)	Necessary (magnetically responsive carriers)	Necessary (microbubbles, nanodroplets, piezo1 or thermosensitive materials)
Operation / Hardware	Light sources and imaging are widely available; deep dosing often needs fibers	Focused ultrasound requires acoustic focusing and thermal monitoring; photothermal needs stable optics	Requires strong gradients or dedicated magnets and navigation	Ultrasound platforms are widely available; require beam planning and monitoring
Off-target mitigation focus	Voxel-level spatial gating and minute-scale shutoff	Focal thermal window restricts exposure; careful cooling to avoid prolonged tails	Field-guided organ or regional enrichment reduces exposure-driven off-targets	Deep, image-guided focal activation with repeatable dosing
Biocompatibility	Minimally invasive overall; potential phototoxicity and heating at high doses; NIR/NIR-II improves safety with careful dosimetry	External stimulus is noninvasive; require temperature control to avoid thermal injury	External stimulus is noninvasive; assess carrier composition and metal burden	External stimulus is noninvasive; constrain cavitation and mechanical index within safety limits
Biomaterials-dependent safety†	Yes (partial strategies)	Yes (common)	Yes (core dependence)	Yes (core dependence)
Limitations	Limited deep-tissue reach; phototoxicity or unintended heating possible; NIR-II hardware and materials raise complexity; fibers or implants add procedural burden	Turn-off limited by cooling; requires real-time thermometry and closed-loop control; photothermal paths inherit optical limits; long-term safety of repeated sub-ablative heating needs evidence	High-precision focusing constrained by gradients and geometry; achieving strong *in vivo* enrichment is difficult; hardware bulk and safety limits apply; carrier persistence may sustain exposure	Beam aberration near bone or air; carrier reliance may introduce secondary activation or retention; rigorous control of thermal and mechanical bioeffects required
Representative refs*	*All citations for each row are provided in the corresponding sections of the main text.			

Notes: Depth and latency values indicate typical orders of magnitude and depend on tissue, dose, and safety windows. NIR-I ≈ 700-900 nm; NIR-II ≈ 1000-1700 nm. † Biomaterials-dependent safety denotes that although the external energy is noninvasive, the system commonly requires functional carriers (nanomaterials, polymers, microbubbles or nanodroplets, photosensitive or thermosensitive parts) delivered locally or intravenously, so immunogenicity, clearance, long-term retention, and tissue reactions directly influence overall biosafety.

## Abbreviations

CRISPR: clustered regularly interspaced short palindromic repeats
Cas9: CRISPR-associated protein 9
DSB: double-strand break
PAM: protospacer adjacent motif
crRNA: CRISPR RNA
tracrRNA: trans-activating CRISPR RNA
sgRNA: single-guide RNA
NHEJ: nonhomologous end-joining
HDR: homology-directed repair
indel: insertion/deletion
nCas9: nickase Cas9
dCas9: catalytically dead Cas9
SpCas9: Streptococcus pyogenes Cas9
ZFN: zinc-finger nuclease
TALEN: transcription activator-like effector nuclease
RNP: ribonucleoprotein
UV: ultraviolet
FRL: far-red light
NIR: near-infrared
NIR-I: near-infrared window I
NIR-II: near-infrared window II
UCNP: upconversion nanoparticle
PEI: polyethylenimine
pSPN: photolabile semiconducting polymer nanotransducer
HSP: heat shock protein
HSE: heat shock element
HSF: heat shock factor
BBB: blood-brain barrier
MENP: magneto-electric nanoparticle
AMF: alternating magnetic field
SDT: sonodynamic therapy
ROS: reactive oxygen species
Treg: regulatory T cell
m6A: N6-methyladenosine
ABA: abscisic acid
DMNB: 4,5-dimethoxy-2-nitrobenzyl
m1A: N1-methyladenosine
PA-rABE: photoactivatable A-to-I RNA base editor
CMP: complementary oligonucleotide
GMP: good manufacturing practice
MRI: magnetic resonance imaging
AI: artificial intelligence
